# Modulating ultralong room-temperature phosphorescence through mechanical confinement of tailored polymer/MOF hybrid interfaces†

**DOI:** 10.1039/d5sc03727a

**Published:** 2025-07-15

**Authors:** Samraj Mollick, Vishal Kachwal, Benjamin Hupp, Yogeshwar D. More, Michele Tricarico, Andreas Steffen, Jin-Chong Tan

**Affiliations:** a Multifunctional Materials & Composites (MMC) Laboratory, Department of Engineering Science, University of Oxford Parks Road Oxford OX1 3PJ UK jin-chong.tan@eng.ox.ac.uk; b Department of Chemistry and Chemical Biology, TU Dortmund Otto-Hahn-Straße 6 Dortmund 44227 Germany

## Abstract

Achieving ultra-long room temperature phosphorescence (RTP) remains a significant challenge due to the inherent trade-off between excited-state lifetime and photoluminescence quantum yield (QY). Herein, we report the synthesis of polymer/metal–organic framework (MOF) hybrids *via* a bottom-up approach, enabling the formation of direct covalent integration between polymer matrices and MOF structures. By systematically optimizing the incorporation of long-chain alkyl amines during the synthesis process, the resultant hybrids demonstrate green RTP performance, achieving a lifetime of 359 milliseconds (ms) at room temperature (592 ms at 77 K), and a phosphorescence QY of ∼28%, whereas no detectable phosphorescence is observed in the parent MOFs and polymer components. Notably, nanoindentation-based mechanical analysis reveals, for the first time, a clear relationship between increased matrix rigidity and enhanced RTP performance. Additionally, a detailed investigation highlights the pivotal roles of extensive intramolecular hydrogen bonding and covalent linking between the polymer and extended frameworks in stabilizing triplet states, enabling efficient RTP. The hybrid materials also demonstrate good processability, allowing the creation of flexible RTP-emissive fibers and films (lifetimes of 408 ms at RT and 613 ms at 77 K) that leverage their inherent flexibility. Furthermore, these hybrids exhibit good selectivity in detecting water over other alcohols, underscoring their potential for smart sensor applications.

## Abstract 2

Achieving ultra-long room temperature phosphorescence (RTP) remains a significant challenge due to the inherent trade-off between excited-state lifetime and photoluminescence quantum yield (QY).

## Graphical abstract

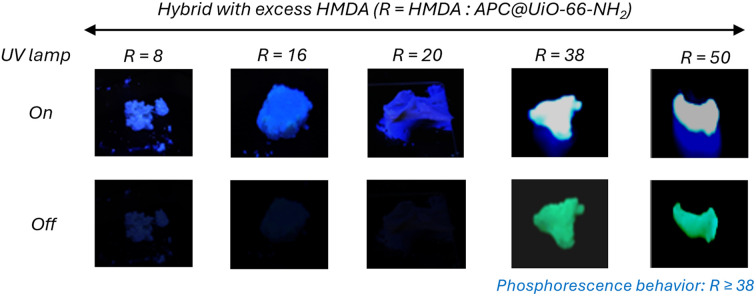

## Introduction

Room temperature phosphorescence (RTP) represents an intriguing phenomenon with extensive applications, ranging from real-time environmental sensing to energy-efficient next-generation lighting, positioning its realization under ambient conditions as an impactful area of exploration.^1–3^ Nonetheless, attaining ultra-long RTP has been a difficult task due to a variety of intrinsic factors. Unlike transition metal complexes, where the presence of heavy atoms facilitates ultrafast intersystem crossing (ISC) through metal-to-ligand charge transfer (MLCT) on the timescale of femtoseconds to picoseconds, organic RTP systems, which lack heavy atoms, present significant challenges. In contrast to their metal-based counterparts, purely organic RTP materials often suffer from inherently inefficient ISC and are dominated by non-radiative decay pathways, which together severely limit their ability to achieve ultra-long RTP under ambient conditions.^4,5^ Furthermore, achieving ultralong phosphorescence lifetimes in such systems is further hindered by vibrational deactivation, oxygen quenching, and dynamic molecular motions, all of which promote excited-state deactivation.^6–9^ To address these challenges and realize the full potential of RTP, it is imperative to concentrate on two fundamental strategies. Initially, it is essential to improve the promotion of singlet to triplet intersystem crossing, which will support the accumulation of long-lived triplet excitons. Simultaneously, it is crucial to concentrate on the reduction of non-radiative quenching processes that facilitate the return of triplet excitons to the ground state, which in turn extends their luminescence lifetime.^10–14^ Moreover, the integration of RTP molecules into a structurally stable framework provides a valuable strategy for reducing non-radiative decay pathways. Mechanical confinement of RTP emitters within a relatively rigid matrix restricts molecular motion and conformational changes, which minimizes the chances for non-radiative deactivation of excited states.^15–19^

Metal–organic frameworks (MOFs) represent a class of hybrid materials that are composed of metal ions or clusters coordinated with multitopic organic ligands, offering a versatile platform for multifunctional applications.^20–24^ Despite the significant interest in luminescent MOFs, their luminescent properties are predominantly characterized by singlet-state fluorescence, which is defined by nanosecond-scale lifetimes.^25–27^ However, MOF-based phosphorescent systems offer distinct advantages over traditional organic phosphors. These benefits include superior photostability and the ability to finely tune emission properties through structural modifications of either the organic linkers or metal nodes. The inherently rigid framework of MOFs effectively restricts molecular motion, thereby minimizing non-radiative losses and enhancing quantum efficiency. Additionally, the incorporation of metal centers promotes intersystem crossing, contributing to improved phosphorescent performance.^28^ The porous nature of MOFs also allows guest molecule encapsulation, offering added functionality and tunability. Regardless of their huge potential, these materials encounter challenges including elevated costs, questionable toxicity profiles, and physical constraints in visible light emission. Moreover, materials that display RTP characteristics are frequently found in powder forms, which presents difficulties for their practical use. To tackle these limitations, there is an increasing focus on the development of polymer/MOF hybrids that merge the functional attributes of MOFs with the adaptability and processability of soft materials such as polymers.^29,30^ This approach facilitates the fabrication of various functional forms that are appropriate for practical use, addressing the challenges linked to the inherently powdery states of MOF-based starting materials.^31,32^ Despite the remarkable potential of polymer/MOF hybrids in a variety of applications, to the best of our knowledge, the pursuit of ultralong RTP in these hybrid systems has not yet been investigated and understood.

This study presents the development of a multifunctional polymer/MOF hybrid that demonstrates extended RTP lifetimes, achieved through the covalent integration of the UiO-66-NH_2_ MOF with polymer matrices. The local chemical environment at the hybrid interface was investigated using state-of-the-art nearfield characterization techniques, namely nanoFTIR and scattering-type scanning near-field optical microscopy (s-SNOM) imaging, shedding light on their distinctive RTP behavior. Exploiting the shaping flexibility of polymer/MOF hybrids, we fabricated monolithic structures, films, and fibers with pronounced RTP characteristics. A thorough nanoindentation-based mechanical investigation uncovered a direct correlation between the structural rigidity of the surrounding matrix and RTP lifetime, a relationship that, although well-theorized, has now been experimentally validated for the first time. Finally, we demonstrated the practical applications of these phosphorescent films and fibre samples for targeted water detection in alcohol mixtures, showcasing one of their potential utilities as a selective luminescent sensor.

## Results and discussion

**Fig. 1:**
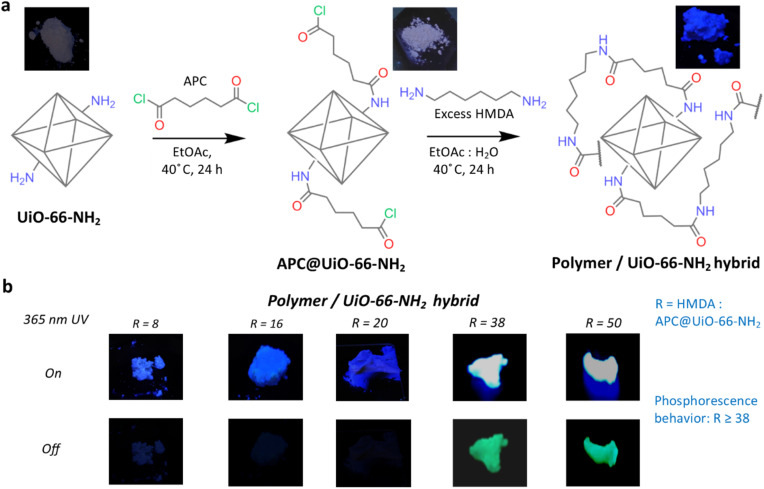
Synthesis pathway and inspection of the photoluminescence behavior of the hybrid materials. (a) Schematic illustration of the synthesis process for the polymer/UiO-66-NH_2_ hybrid material. Abbreviations: APC, adipoyl chloride; HMDA, hexamethylene diamine; EtOAc, ethyl acetate. Photoluminescence images (top insets) depict the solid-state emission of UiO-66-NH_2_, APC@UiO-66-NH_2_, and the polymer/UiO-66-NH_2_ hybrid under 365 nm UV light. (b) Photoluminescence images of polymer/UiO-66-NH_2_ hybrids with varying ratios (R) of HMDA-to-APC@UiO-66-NH_2_. The ‘On’ state corresponds to excitation under the UV lamp (365 nm), while the ‘Off’ state corresponds to an absence of UV excitation. Phosphorescence behavior is observed when *R* ≥ 38, as indicated by the extended luminescence upon the switching off of the UV lamp.

The polymer/MOF hybrid was synthesized by a rationally designed two-step process (Fig. 1a). Initially, the amine-functionalized MOF, UiO-66-NH_2_, underwent covalent modification with adipoyl chloride (APC), resulting in the formation of the APC-functionalized UiO-66-NH_2_ MOF (APC@UiO-66-NH_2_). A polymerization reaction was subsequently initiated with an excess of hexamethylene diamine (HMDA) in relation to the MOF, designated as *R* = HMDA: APC@MOF, where *R* = 8, 16, 20, 38, 50. This second step enabled the formation of a polymer-embedded MOF hybrid through the anchoring of polymeric chains onto the APC-functionalized framework. The luminescent properties of the hybrid material were significantly influenced by the HMDA content during the second step of synthesis (Fig. 1b and S1†). A remarkable enhancement in photophysical performance was observed with increasing amine content, reaching a plateau when the HMDA-to-APC@MOF ratio *R* has surpassed 38. It is noteworthy that RTP was exclusively observed in hybrids with *R* ≥ 38. This critical ratio seems to indicate the beginning of extensive intramolecular and supramolecular interactions within the hybrid framework, which are crucial for stabilizing the triplet states and facilitating efficient phosphorescence (Fig. 1b). Considering this threshold behavior, hybrids synthesized with *R* = 38 were selected for all subsequent characterization and functional evaluations to study the optimal luminescence performance and reproducibility.

**Fig. 2:**
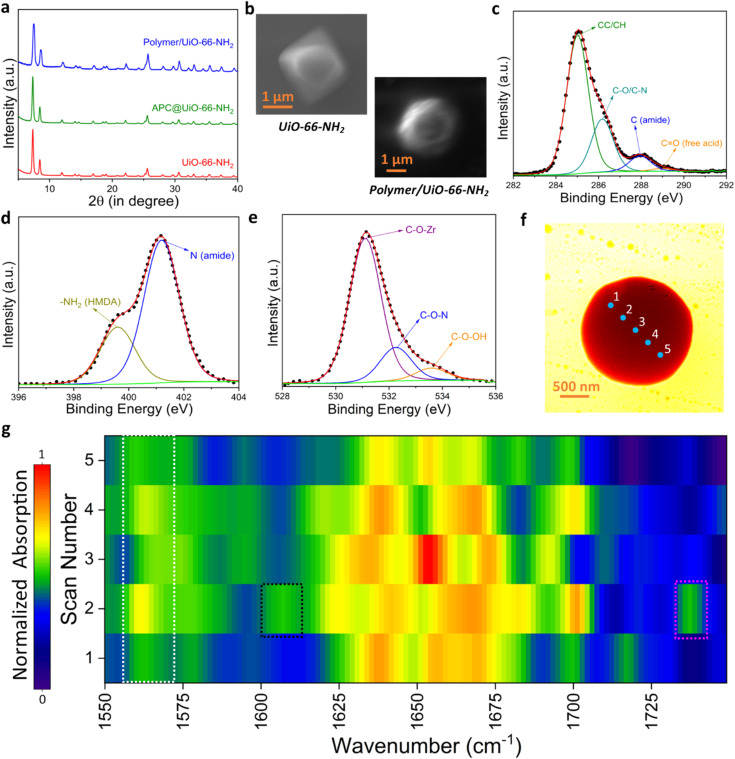
Characterization of the polymer/UiO-66-NH_2_ hybrid (*R* = 38) and related materials. (a) PXRD patterns of UiO-66-NH_2_, APC@UiO-66-NH_2,_ and the polymer/UiO-66-NH_2_ hybrid. (b) FESEM micrographs of pristine UiO-66-NH_2_ and the polymer/UiO-66-NH_2_ hybrid. XPS spectra of the hybrid: (c) C 1s, (d) N 1s, and (e) O 1s. (f) Nearfield infrared white light image of the polymer/UiO-66-NH_2_ hybrid. (g) NanoFTIR absorption spectra of the polymer/UiO-66-NH_2_ hybrid crystal measured point-by-point (at spatial resolution ∼20 nm) across the locations highlighted in (f).

A comprehensive structural characterization of the materials was carried out using multimodal techniques, including powder X-ray diffraction (PXRD), scanning electron microscopy (SEM), nuclear magnetic resonance (NMR), X-ray photoelectron spectroscopy (XPS), Fourier transform infrared spectroscopy (FT-IR), and near-field infrared nanospectroscopy (Fig. 2). The PXRD studies validated the preservation of the bulk phase purity and crystallinity of the MOF host, even following the polymerization process over MOFs (Fig. 2a).

SEM analysis was performed to examine the morphological characteristics of the resulting materials (Fig. 2b and S2†). The SEM images demonstrated that the pristine UiO-66-NH_2_ crystals exhibited a diamond shape, measuring around 1.5–1.6 μm in size. After the polymerization process, the crystals exhibited a transformation into a spherical shape with an increased size (∼2.86 μm), indicating successful polymerization even on the MOF crystal surface. ^1^H NMR spectroscopy was utilized to examine the extent of amine functionality and the covalent connection between the MOF host and polymers (Fig. S3 and S4†). ^1^H NMR analysis of digested samples revealed the presence of amide groups within the composites (∼9 ppm), which were formed through two pathways: the condensation of MOF-bound amines with adipoyl chloride, and the reaction between HMDA and adipoyl chloride (Fig. S4a†). The distinct peaks observed for amine-condensed adipoyl chloride (∼2.3 ppm and ∼1.5 ppm) confirmed the successful covalent linkage between the amine functionalities of the MOF and adipoyl chloride. With increasing concentrations of HMDA, there was a noticeable decrease in the aforementioned peaks, indicating that the excess HMDA was obscuring them. Moreover, the peaks observed at 2.8 ppm corresponding to –CH_2_ groups confirmed the substantial amount of HMDA present in the composites (Fig. S4b†).

To gain a deeper insight into the surface of the polymer/MOF hybrid (*R* = 38), XPS analysis was performed (Fig. 2c–e, S5 and S6†). A significant shift in the Zr 3d peaks was noted, with a movement towards lower binding energy (3d_5/2_: 183.1 eV to 182.4 eV; 3d_3/2_: 185.4 eV to 184.9 eV), suggesting an increase in electron density across the MOF, likely due to the surplus of HMDA incorporated into the materials (Fig. S5†). The identification of the amide group at 286.1 eV in the C 1s spectrum confirms the hybridization taking place between the amine group of the MOF and the carboxyl group from APC (Fig. 2c). Additionally, the carbonyl peak detected at 288.9 eV validates the presence of unreacted acid groups originating from APC. A new peak at 399.6 eV in the N 1s spectra indicates the presence of primary alkyl amine groups, thereby confirming the excess of HMDA in the composite (Fig. 2d and S6c†). Furthermore, the amine functionality of the MOF, initially detected at 400.1 eV, showed a shift to 401.1 eV, indicating the formation of amides and suggesting a covalent bond between the polymer and the MOF framework (Fig. S4b†). The O 1s peak associated with C–O–Zr showed a shift to lower energy (from 532.0 eV to 531.1 eV) after polymerization, providing additional evidence of the interaction between the polymer and MOF (Fig. 2e and S6b†). The appearance of a new amide-related O 1s peak at 532.3 eV offers further support for the formation of a polymer-framework hybrid structure (Fig. 2e and S5b†). This comprehensive XPS analysis offers strong support for the successful integration as well as chemical bonding between the polymer and MOF in the composite.

The ATR-FTIR spectra of the phosphorescent materials provided important insights into the molecular structure of the polymer-MOF hybrid composites (Fig. S7†). The analysis of the spectra from pristine MOFs and their intermediate products provided substantial evidence indicating that polymerization is successfully taking place within the MOF host framework. Bulk FTIR analysis demonstrated distinct carbonyl peaks on the benzene ring at 1383 cm^−1^ and 1566 cm^−1^, along with the NH_2_ functional group vibrations at 1654 cm^−1^, which are characteristic indicators for MOFs.^33^

### Local area investigation of nanosized particles

State-of-the-art nearfield probing techniques such as s-SNOM imaging and nanoFTIR were employed to delve deeper into the interface between the polymer and MOF at the nanoscale level (spatial resolution of ∼20 nm) within the sub-micron crystal (Fig. 2f, g and S8†).^34,35^ The vibrational peak observed at around 1560 cm^−1^ (white highlighted band in Fig. 2g) confirms that the framework structure of the MOF host has remained intact at the local nanoscale region during the polymer growth process. Additionally, the appearance of new peaks linked to the amide groups –C–N around 1546 cm^−1^ and various types of secondary amides around 1625–1670 cm^−1^ highlighted the direct covalent connection between the amine functionalities of the MOF and the polymer chains, detected at the nanoscale, offering further proof of the formation of a stable polymer-MOF network. A peak at 1607 cm^−1^ (black highlighted region in Fig. 2g) linked to free NH_2_ groups indicates that excess amine functionalities remained in the material, likely contributing to its unique chemical properties. Additionally, the detection of a free carboxylic acid peak at 1735 cm^−1^ (pink highlighted region in Fig. 2g) confirms the existence of free carboxylic acid in the materials. The hypothesis suggests that the unreacted APC experienced hydrolysis throughout the polymerization process, leading to the generation of free acidic groups. This nanoscale observation underscores the intricate chemistry taking place at the vicinity of the MOF-polymer interface; nonetheless, a more detailed structural analysis is required to properly evaluate the degree of unreacted APC and its impact on the overall properties of the composite.

To study the electronic transitions in the polymer-MOF hybrid, we conducted solid-state UV-vis measurements, which provided valuable insights into the evolving optical properties upon polymerization (Fig. S9†). Our investigation revealed that, following polymerization over MOFs, there is a notable broadening of the absorption peak from 400 to 550 nm, a feature that is absent in the pristine MOF samples. The prominent peaks noted can be linked to transitions that may arise from the interactions between the lone pairs of electrons on the oxygen and nitrogen atoms within the polymer matrix and the antibonding π* orbitals of the carbonyl groups in the hybrid, facilitating the *n* → π* transitions. Another probability of intramolecular charge transfer (ICT) arises from the effective charge transfer between the polymer (rich in amide and diamine groups) and the MOF ligands creating new electronic states, evidenced as broader and lower-energy absorption features.

### Correlation between phosphorescence and polymer rigidity

**Fig. 3:**
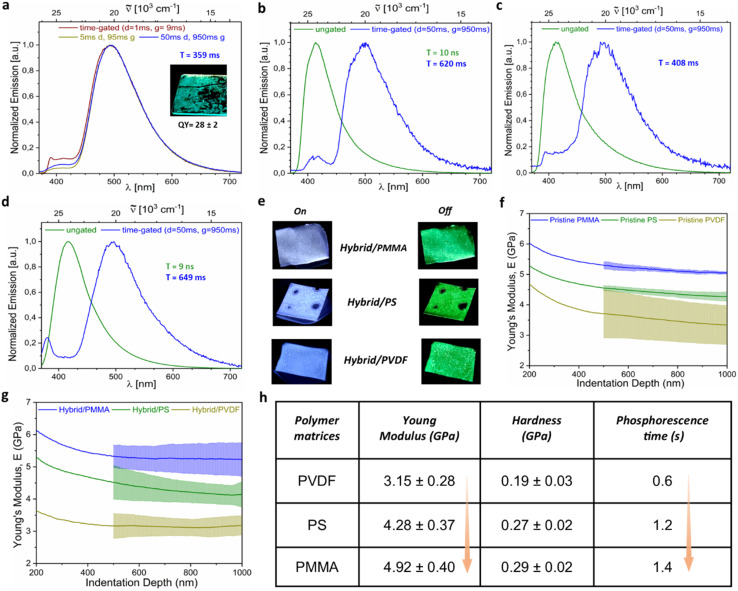
Luminescent and mechanical properties of polymer/UiO-66-NH_2_ hybrid (*R* = 38) materials. (a) Gated photoluminescence (PL) spectra of the polymer/UiO-66-NH_2_ hybrid solid powder at room temperature (RT), with the inset showing solid-state green phosphorescence after brushing the powder onto a glass substrate. The phosphorescent quantum yield (QY in %), and average lifetime (T). (b) Ungated and gated PL spectra of the solid powder at 77 K. (c and d) Comparison of ungated and gated PL spectra of PMMA films embedded with the polymer/UiO-66-NH_2_ hybrid at (c) RT and (d) 77 K. (e) Photographs of various films embedded with the polymer/UiO-66-NH_2_ hybrid under UV light (365 nm, ‘On’) and without UV light (‘Off’), showcasing luminescence. (f and g) Nanoindentation analysis of films, illustrating (f) the average indentation modulus and indentation depth of pristine films and (g) those embedded with the polymer/UiO-66-NH_2_ hybrid. (h) Tabulated correlations between Young's modulus, hardness, and phosphorescence time (see movies) across composite films comprising hybrids embedded into various polymeric matrices, demonstrating the interplay of mechanical behavior and luminescent properties.

We systematically investigated the photoluminescent (PL) properties of the polymer/MOF hybrids, thereby systematically revealing their tunable luminescence behavior (Fig. 3 and S10–S13†). The pristine polymer, MOF (UiO-66-NH_2_), and physical mixing of the MOF and polymer are weakly luminescent (Fig. S10† and 1a). The pristine MOF displayed emission peaks at 452 nm, while APC@MOF exhibited emission peaks at 468 nm, with additional shoulders at 413 nm and 516 nm upon excitation at 350 nm (Fig. S11†). However, the fluorescence quantum yield (QY) for these materials remained below 3% (Fig. S1†). Fluorescence lifetime measurements of the hybrid indicated a short average lifetime of 10 ns, confirming the dominant fluorescence behavior (fluorescent QY ∼38%). Interestingly, composites with *R* ≥ 38 exhibited clear RTP upon 365 nm UV excitation, while lower ratios did not show any such phosphorescence behavior (Fig. 1b). This prompted a detailed study of the phosphorescence behavior using gated measurements to isolate long-lived emissions from the faster fluorescence. The gated PL spectra demonstrated a phosphorescence emission maximum at 494 nm, with only a minor fluorescence shoulder at 420 nm (Fig. 3a). The ultra-long phosphorescence lifetime of 359 ms at room temperature confirmed the emission at 494 nm as phosphorescence (Fig. 3a). The phosphorescence QY of the hybrid (*R* = 38) powder sample is ∼28 ± 2 (Fig. 1a).

Upon cooling to 77 K, the phosphorescence lifetime of the polymer/MOF hybrids significantly increased from 359 ms to 592 ms (Fig. 3b and S13†).^36^ This enhancement underscores the suppression of molecular vibrations at lower temperatures, reducing non-radiative and enhanced radiative decay. Since the hybrids incorporate excess flexible alkyl chains from HMDA, their molecular vibrations contribute to non-radiative decay pathways. To mitigate this, the hybrids were embedded into polymer matrices of varying mechanical stiffnesses, including polyvinylidene fluoride (PVDF), polystyrene (PS), and polymethyl methacrylate (PMMA) (Fig. 3e–h and S12†). Mechanical properties such as Young's modulus (*E* is a measure of stiffness) and hardness were characterized, showing PMMA as the stiffest matrix (*E* = 5.16 GPa), followed by PS (*E* = 4.41 GPa) and PVDF (*E* = 3.64 GPa) (Fig. 3f and S14†).^34^

Upon embedding the phosphorescent hybrid into the polymer matrix, we observed a consistent reduction in the mechanical stiffness of all composites due to the soft nature of the hybrids (Fig. 3g and h). Despite this decrease, a clear correlation emerged between matrix stiffness and phosphorescence lifetime. As Young's modulus increased from 3.15 GPa (PVDF films) to 4.92 GPa (PMMA films), the RTP lifetime proportionally extended from 0.6 seconds to 1.4 seconds (Fig. 3h, see movies). Similarly, the hardness values (0.19–0.29 GPa) mirrored this trend, with PMMA-embedded hybrids demonstrating the longest lifetimes (359 ms for the bare composite extended to 408 ms). Notably, at 77 K, we observe a bi-exponential decay with lifetimes of 155 ms and 661 ms, with an average lifetime of 613 ms. Hence, the lifetimes of the PMMA-embedded polymer/MOF hybrid further increased under liquid nitrogen conditions from 408 ms to 613 ms (Fig. 3d), where molecular vibrations were effectively frozen, minimizing non-radiative decay. These findings highlight the pivotal role of matrix stiffness in controlling the phosphorescence behavior of hybrid materials. Enhanced stiffness restricts molecular vibrations, suppressing non-radiative decay pathways and stabilizing the triplet state, thereby extending phosphorescence lifetimes. To the best of our knowledge, this systematic study is the first for the MOF-based hybrid to establish a direct relationship between polymer matrix mechanical properties and phosphorescence lifetime, offering new insights into material design for advanced photonic applications.

**Fig. 4:**
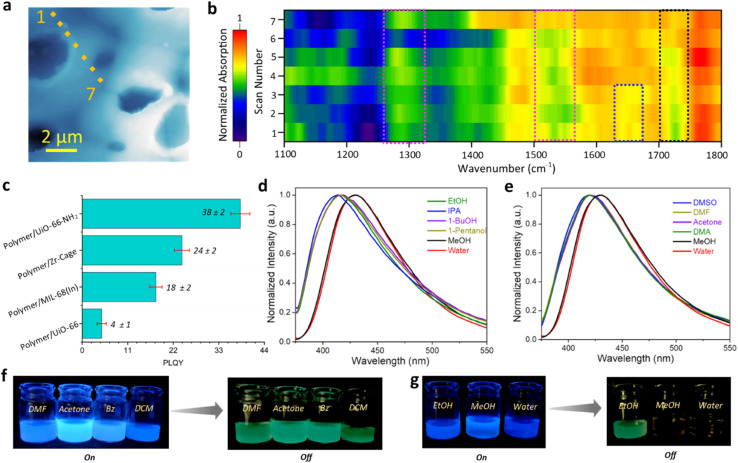
Characterization of the polymer/UiO-66-NH_2_ hybrid (*R* = 38) in PVDF films and investigations into the phosphorescence mechanism of the polymer/framework hybrids. (a) Specific positions marked on the image corresponding to the nanoFTIR scans on the local regions of the hybrid/PVDF film, with a probe size of ∼20 nm. (b) NanoFTIR absorption spectra of a hybrid/PVDF film taken across the highlighted region in (a). (c) Fluorescence QY of different polymer/framework hybrids. (d) and (e) PL spectra of the hybrid in (d) polar protic solvents and (e) polar aprotic solvents, demonstrating the solvent-dependent behavior. (f) and (g) Photographs of the hybrid material exposed to different solutions under UV light (“On”) and in the absence of UV light (“Off”), showcasing variations in luminescence response. The hybrid displays phosphorescence behavior in a variety of solvents, excluding methanol and water.

The (conventional) far-field FTIR and near-field nanoFTIR characterization of PVDF-embedded hybrids confirmed the retention of the hybrid's core structure and composition (Fig. 4a, b and S15†). Distinct spectral features were observed: the pink-highlighted region in Fig. 4b corresponds to the characteristic signals of the MOF framework, the blue-highlighted region denotes secondary amine groups formed *via* the condensation reaction between amines and APC, and the black-highlighted region indicates free acid groups resulting from the hydrolysis of APC during the polymerization process. These findings affirm the structural stability of the hybrid materials embedded within the polymer matrix film.

### Mechanistic insight into the phosphorescence behavior

To unravel the key requirements for inducing phosphorescence in polymer-framework hybrid materials, a systematic series of controlled experiments was undertaken, evaluating both structural and chemical parameters (Fig. 4c–g and S16†). The initial investigation examined the impact of framework functionalization by polymerizing on UiO-66, a Zr-based MOF lacking amine functionalities. The complete absence of phosphorescence in this system highlighted the necessity of hybridization between the polymer and an amine-functionalized MOF scaffold to yield phosphorescent behavior (Fig. S16b†). Subsequently, polymerization was conducted on discrete Zr-cage molecules functionalized with amine groups, but lacking the extended connectivity of a framework (Fig. 4b).^37^ While the resulting material exhibited enhanced photoluminescent properties, it failed to demonstrate phosphorescence (Fig. S16c†). This result indicates that luminescence enhancement alone is insufficient; an extended framework structure is crucial for stabilizing the triplet states, a prerequisite for phosphorescence. In a parallel study, polymerization was carried out on MIL-68(In)–NH_2_, a MOF with a one-dimensional channel-like architecture and significantly larger pores than UiO-66-NH_2_.^38^ These materials also failed to exhibit phosphorescence, reinforcing that only densely packed, amine-functionalized frameworks, such as UiO-66-NH_2_, offer the requisite combination of strong intramolecular hydrogen bonding and structural confinement necessary to effectively stabilize triplet excitons and enable persistent phosphorescence.

To gain a deeper insight into the RTP mechanism, we investigated the effect of solvent environments by dispersing the hybrid composites in solvents with varying polarities, including polar protic, polar aprotic, and nonpolar solvents (Fig. 4e–g). Remarkably, while phosphorescence was stable in most solvents, it diminished significantly in methanol and water. This behavior is attributed to strong hydrogen-bonding interactions between these solvents and the polymer/MOF hybrid, which disrupt the intramolecular hydrogen-bonding network crucial for sustaining RTP.

Conversely, in other solvents, where such disruption was minimal, the phosphorescence behavior persisted, demonstrating the importance of maintaining a robust hydrogen-bonding network within the polymer matrix of the hybrid. Overall, these findings reveal that achieving RTP in polymer/MOF hybrids requires a synergistic combination of extended framework hybridization and strong intramolecular interactions, facilitated by abundant amine functionalities such as those provided by HMDA. These insights establish a foundational understanding of the hybrid systems' structural and chemical parameters that are underpinning the phosphorescent response of hybrid systems.

### Applications

**Fig. 5:**
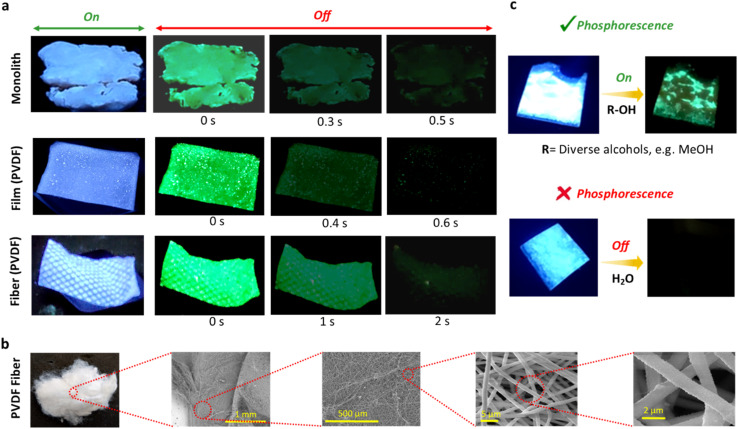
Applications of the polymer/UiO-66-NH_2_ hybrid (*R* = 38) in diverse forms and functional tests. (a) Various hybrid forms, including monolith, film (hybrid embedded within a PVDF matrix), and fiber (constructed from the hybrid and PVDF matrix). (b) FESEM micrographs of fibers comprising the polymer/UiO-66-NH_2_ hybrid and PVDF matrix, highlighting the porous structural morphology. (c) Water-sensing application using a PS film embedded with the polymer/UiO-66-NH_2_ hybrid. Photographs illustrate that the film exhibits phosphorescence in various alcohols (such as methanol, ethanol, isopropanol, and 1-butanol), while no phosphorescence is observed in water, demonstrating its selective sensing behavior.

The versatile and malleable nature of the phosphorescent hybrids enabled their fabrication into diverse morphologies, including monoliths, films, and fibers, to investigate the impact of material form on phosphorescent performance (Fig. 5 and Movies S1–S4†). Among these, fibers demonstrated the longest phosphorescence lifetimes, surpassing the films, despite both incorporating the PVDF matrix (Fig. 5a, Movies S1 and S3†). This enhanced lifetime in fibers is attributed to their highly ordered structure, which facilitates molecular alignment and tighter packing (Fig. 5b).^39,40^ Such confinement effectively reduces non-radiative decay pathways, thereby preserving triplet states and minimizing energy dissipation. To our knowledge, this is the first instance of utilizing a polymer/MOF hybrid for fiber fabrication. Alongside their prolonged lifetimes, films and fibers exhibited improved photostability compared to monoliths (Fig. S17†). This is because phosphorescent hybrids are protected in the polymeric matrices in films and fibers.

This advantage arises from the protective role of the polymer matrix, which shields the embedded phosphorescent materials from photodegradation during UV exposure. These findings underscore the importance of morphology in optimizing both the photophysical and stability properties of hybrid materials. Beyond photostability and lifetime measurements, these materials were explored for selective solvent detection, particularly focusing on water and alcohol (Fig. 5c, S18, S19, and Movies S4–S7†). It was observed that the hybrid's powder alone exhibited no phosphorescence in both methanol and water (Fig. 4f and g). In contrast, the hybrid/PS films retained their phosphorescent behavior in methanol (Movies S4 and S5†). This persistence is attributed to the polymer matrix's ability to protect the hybrid material from methanol-induced disruptions, thereby preserving the critical intramolecular hydrogen bonding essential for phosphorescence. However, in the presence of water, the hybrid/PS films and hybrid/PVDF fiber do not exhibit any phosphorescence (Fig. 5c, S19, Movies S6 and S7†). This behavior is due to the water molecules penetrating the materials, disrupting intramolecular hydrogen bonding, and forming stronger intermolecular hydrogen bonds, subsequently quenching their phosphorescence. Importantly, the materials retained their phosphorescent behavior in all other solvents tested, further demonstrating their chemical selectivity. This unique response to water highlights the potential of these phosphorescent hybrids for water detection applications, providing a robust and sensitive platform for environmental monitoring and solvent identification.

## Conclusions

In summary, we successfully demonstrated ultralong RTP in polymer/MOF hybrids, achieving a lifetime of up to 359 ms (powder sample) and a phosphorescence QY of ∼28% under ambient conditions through direct covalent hybridization between polymer matrices and MOFs. These results indicate a substantial improvement over pristine MOFs and neat polymers, both of which exhibit no detectable RTP. Importantly, our study provides the first experimental evidence of a direct correlation between the modularity of structural rigidity in hybrids and RTP lifetimes, establishing a fundamental design principle for developing advanced phosphorescent materials. The mechanical properties of these hybrids were thoroughly characterized using nanoindentation techniques, revealing that enhanced structural rigidity effectively suppresses non-radiative decay pathways, thereby stabilizing the triplet state and extending phosphorescence durations. Furthermore, we demonstrated the versatility of these hybrid materials by fabricating various functional forms, including flexible fibers, showcasing their potential for integration into next-generation optoelectronics and sensing devices. This work underscores the transformative potential of polymer/MOF hybrids in creating multifunctional, processable RTP materials, paving the way for breakthroughs in tunable luminescent technologies.

## Author contributions

S. M. conceived and designed the project. S. M., V. K., and Y. D. M. carried out the synthesis of all materials and performed data analysis. S. M. conducted nanoFTIR and analyzed the data with the help of J. C. T., M. T. and S. M. conducted the nanoindentation experiments and analyzed the results. B. H. and A. S. carried out phosphorescence measurements and data analysis. J. C. T. supervised the project. All authors discussed the results and contributed to the final version of the manuscript.

## Conflicts of interest

The authors declare no conflict of interest.

## Supplementary Material

SC-016-D5SC03727A-s001

SC-016-D5SC03727A-s002

SC-016-D5SC03727A-s003

SC-016-D5SC03727A-s004

SC-016-D5SC03727A-s005

SC-016-D5SC03727A-s006

SC-016-D5SC03727A-s007

SC-016-D5SC03727A-s008

## Data Availability

The data supporting this article have been included as part of the ESI.†
