# Supplementary material for: Modulating ultralong room-temperature phosphorescence through mechanical confinement of tailored polymer/MOF hybrid interfaces
Source: Chem Sci. 2025 Jul 15;16(33):15185–93. doi: 10.1039/d5sc03727a (PMC12285017; doi:10.1039/d5sc03727a)
Supplement: SC-016-D5SC03727A-s001 [file SC-016-D5SC03727A-s001.pdf]

This journal is © The Royal Society of Chemistry 2025

Supplementary Information

Modulating Ultralong Room-Temperature Phosphorescence Through Mechanical Confinement of Tailored Polymer/MOF Hybrid Interfaces

Samraj Mollick[a], Vishal Kachwal[a], Benjamin Hupp[b], Yogeshwar D. More[a], Michele Tricarico[a], Andreas Steffen[b], Jin-Chong Tan[a]*

[a]Multifunctional Materials & Composites (MMC) Laboratory, Department of Engineering Science, University of Oxford, Parks Road, Oxford OX1, United Kingdom.

[b]Department of Chemistry and Chemical Biology, TU Dortmund University, Otto-Hahn-Straße 6, 44227 Dortmund, Germany.

*Corresponding author's e-mail: jin-chong.tan@eng.ox.ac.uk

Table of Contents

Materials	S-2
Materials synthesis	S-2 to S-4
Materials characterization and physical measurement techniques	S-5 to S-7
Structural characterization of hybrid	S-8 to S-15
Optical characterization	S-16 to S-19
Mechanical properties	S-20
Controlled tests	S-21
Applications	S-22 to S-23
Movie clips	S-24
References	S-25

Materials

All the reagents, solvents, and photochromic guests were commercially sourced and used as received. All of the commercially available materials were purchased from Sigma-Aldrich. Fisher Scientific, and Fluorochem depending on their availability.

Methods

Materials Synthesis

UiO-66-NH_2. The synthesis of the UiO-66-NH_2 followed a slightly modified procedure based on a previously reported method.[1] To synthesize UiO-66-NH_2, 2-amino terephthalic acid (668 mg, 3.69 mmol) and $ZrCl_4$ (233 mg, 1 mmol) were dissolved in 50 mL of N, N-dimethylformamide (DMF) within a tightly sealed glass bottle (100 mL) and subjected to ultrasonication for 10 minutes. Subsequently, 5 mL of acetic acid was introduced into the mixture, and the reaction vessel was heated at 130 °C for 48 hours. The system was then allowed to cool gradually to room temperature over 12 hours. The resultant yellow solid was isolated via centrifugation, thoroughly washed with excess DMF and methanol, and soaked in methanol for five days with daily solvent replacement. Finally, the material was dried under vacuum at 120 °C for 12 hours.

UiO-66. The synthesis of UiO-66 involved combining terephthalic acid (613 mg, 3.69 mmol) with $ZrCl_4$ (233 mg, 1 mmol) in 50 mL of DMF in a sealed glass bottle (100 mL), followed by ultrasonication for 10 minutes. Acetic acid (5 mL) was added, and the mixture was heated at 130 °C for 48 hours. The system was cooled slowly to room temperature over an additional 12-hour period. The resulting colorless powder was collected by centrifugation, washed extensively with DMF and methanol, soaked in methanol for five days with daily solvent exchange, and dried under vacuum at 120 °C for 12 hours.

MIL-68(In)-NH_2. 2-amino terephthalic acid (230 mg, 1.3 mmol) and indium nitrate hydrate ($In(NO_3)_3 \cdot xH_2O$, 1490 mg, 3.8 mmol) were dissolved in 12.5 mL of DMF in a tightly capped 20 mL glass vial. The mixture was ultrasonicated for 15 minutes and then heated at 120 °C for 48 hours in an oven. The reaction mixture was cooled gradually to room temperature over 12

hours. The resulting product was separated by centrifugation, washed thoroughly with DMF and methanol, and dried under vacuum at 120 °C for 12 hours.

Zr-Cage. The synthesis of the Zr-Cage followed a slightly modified procedure based on a previously reported method.[2, 3] In this process, 2-aminoterephthalic acid (50 mg, 0.3 mmol) and zirconocene dichloride (150 mg, 0.5 mmol) were dissolved in 10 mL of N,N-dimethylacetamide (DMA) containing a trace amount of water (approximately 40 drops). The mixture was allowed to stand undisturbed at room temperature for 72 hours to facilitate crystal growth. The resulting yellow cubic block crystals were carefully separated by decanting the solution and subsequently dried under vacuum conditions. This approach yielded high-quality crystalline Zr-Cage structures suitable for further study.

Polymer. Adipoyl chloride (APC) acid and hexamethylene diamine (HMDA) were mixed in a 1:12 ratio within a 1:1 solution mixture of water and ethyl acetate. The mixture was stirred continuously for 24 hours at room temperature to ensure thorough polymerization. After the reaction was complete, the solution was decanted to separate the product, which was subsequently dried at 40 °C over two days to obtain the final polymer.

APC@MOF. UiO-66-NH_2 (0.54 mmol, 160 mg) was dispersed in 30 mL of ethyl acetate solution through ultrasonic treatment for 10 minutes. Adipoyl chloride (APC, 6.8 mmol, 1 mL) was then added to the dispersion, and the mixture was stirred at 40 °C for 24 hours. A color change of the MOF particles to a lighter yellow indicated the progress of the reaction. This method was adapted for other framework materials, including MIL-68(In)-NH_2, UiO-66, and Zr-Cage, maintaining a consistent MOF-to-APC molar ratio of 1:12 across all samples. These APC-functionalized MOFs (APC@MOF) served as intermediates for the subsequent synthesis of polymer/MOF hybrid materials.

Polymer/MOF hybrid. A solution of HMDA (6.8 mmol) in 5 mL of water was mixed with a 20 mL vial containing APC@MOF (160 mg) dispersed in 5 mL of ethyl acetate solution and stirred for 24 hours at 50 °C to facilitate polymerization. After completion, the product was separated by decanting the residual solution and subsequently dried in the reaction vial at 40 °C for a couple of days. To obtain hybrid materials with varying compositions, different HMDA-to-APC@MOF ratios (R) were employed. For UiO-66-NH_2 MOF, the ratios R = 8, 16, 20, 38 and 50 were used. In the case of UiO-66, discrete Zr-Cage molecules, and MIL-68(In)-NH_2 MOFs, the synthesis was performed with a fixed ratio of R = 38. This systematic variation

allowed for a tailored investigation of the relationship between the hybrid composition and its properties.

Fabrication of thin films. A variety of thin films was fabricated using various polymer matrices, including polyvinylidene fluoride (PVDF), polystyrene (PS), and polymethyl methacrylate (PMMA). The synthesis began with the dissolution of solid polymer powders in DMF to prepare polymer solutions. For phosphorescent thin films, polymer/MOF hybrids were uniformly dispersed into the polymer matrices through ultrasonication for 20 minutes, followed by magnetic stirring at room temperature for 96 hours. The resulting polymer-nanocomposite solutions were then cast onto glass substrates using the doctor blade method. Films with a thickness of approximately 380-400 μm were produced, maintaining a consistent casting speed of 12 mm/s across all samples. The filler loading of the polymer/MOF hybrid in each film was fixed at 5 wt.%.

Fabrication of fibers. PVDF fibers were synthesized via the electrospinning technique, involving a multistep process. First, PVDF polymer solution was created by dissolving PVDF powder in a 3:1 solvent mixture of DMA and acetone. Next, an electrospinning solution was prepared by blending polymer/MOF hybrid powders with 13.7 wt.% PVDF polymer solutions for 24 hours in stirring condition at room temperature to ensure homogeneous dispersion. To achieve fibers with a filler loading of 5 wt.%, the polymer/MOF hybrid powder was added in a proportion of 5 wt.% relative to the total polymer content. The electrospinning process parameters used to fabricate the fibers are detailed in the subsequent section.

Electrospinning: Electrospinning was carried out at 10.5 kV using a DC high-voltage generator.[4] The PVDF solution was supplied to a G19 needle emitter (nozzle) with a blunt tip *via* a syringe pump at a processing rate of at an infusion rate of 0.40-0.80 mL h^{-1}. The distance from the collector (aluminum foil) to the tip was 17 cm. The aluminum foil attached on a bottom steel plate was used as a collector for the electrospun microfibers (MF).

Materials characterization and physical measurement techniques

Powder X-ray diffraction (PXRD). Powder X-ray diffraction (PXRD) measurements were carried out using a Rigaku MiniFlex diffractometer equipped with a Cu Kα radiation source (λ = 1.541 Å). The scans were performed at a speed of 0.05°/min with a step size of 0.005° in the 2θ range.

Attenuated total reflectance Fourier transform infrared spectroscopy (ATR-FTIR). ATR-FTIR spectra were acquired at ambient conditions using a Nicolet iS10 FTIR spectrometer fitted with an ATR sampling accessory.

X-ray photoelectron spectroscopy (XPS). X-ray photoelectron spectroscopy (XPS) analyses were performed on a Kratos AXIS Ultra DLD system employing monochromated Al Kα radiation as the X-ray source. A charge neutralizer and a hemispherical energy analyzer were used, with pass energies of 160 eV for survey scans and 55 eV for high-resolution elemental scans. Samples were mounted on adhesive carbon tape within a glovebox and transferred into the XPS chamber via a vacuum transfer vessel to prevent ambient contamination.

Near-field nanospectroscopy. Atomic force microscopy (AFM) height topography and near-field infrared nanospectroscopy (nanoFTIR) were conducted using an s-SNOM instrument (Neaspec GmbH). A platinum-coated AFM probe (Arrow-NCPt, tip radius < 25 nm, 285 kHz) was operated in tapping mode and illuminated with a broadband mid-infrared (MIR) laser source (Toptica). NanoFTIR absorption spectra were acquired by modulating the signal at the second harmonic to minimize background noise. Spectra were averaged over 12 individual measurements, with an integration time of 14 seconds each, and normalized to the silicon substrate spectrum. Instrument noise was reduced by removing 200 cm^{-1} of data from both ends of the spectra during plotting.

Scanning electron microscopy (SEM). Morphological characterization of crystals and fibers was conducted using field-emission scanning electron microscopy (FESEM) on a LYRA3 GM TESCAN system and a Hitachi TM3030 Plus 0865 scanning electron microscope.

Diffuse reflectance spectroscopy (DRS). Diffuse reflectance UV-Vis absorption spectra were measured using a Shimadzu 2600 UV-Vis spectrophotometer. Photoluminescence images were captured under ultraviolet light (365 nm) using a handheld UV lamp (6 W).

Solid-state photoluminescence spectra. All photophysical measurements of samples such as UiO-66-NH_2, APC@UiO-66-NH_2, and the hybrid material with R = 38 were performed in the solid state or in a polymer matrix (PMMA, polystyrene, PVDF). Unless specified, experiments were carried out under an ambient atmosphere.

Excitation and emission spectra were recorded on an Edinburgh Instrument FS5 spectrometer, equipped with a 150 W Xenon arc lamp, single monochromators for the excitation and emission pathways, and a photomultiplier (R928P) as detector. The excitation and emission spectra were corrected using the standard corrections supplied by the manufacturer for the spectral power of the excitation source and the sensitivity of the detector. The fluorescence lifetimes were recorded using an EPLED (363.2 nm with 1.7 μW, pulse width 927.3 ps), with a repetition rate of up to 10 MHz, dependent on the time range, and a time-correlated single photon counting (TCSPC) module. The emission was collected at a right angle to the excitation source. The phosphorescence duration referenced in Figure 3h of the main manuscript was determined through analysis of the supplementary videos, as detailed on page S-24 of this supporting information file.

Time-gated excitation and emission spectra, as well as 77 K data, were recorded on an Edinburgh Instrument FLS1000 spectrometer, equipped with a 450 W Xenon arc lamp for steady-state experiments and a μF2 pulsed 60 W Xenon microsecond flashlamp for time-gated experiments, double monochromators for the excitation and emission pathways, and a red-sensitive photomultiplier (PMT-980) as detector.

For time-gated experiments, both the delay d (time period between the light pulse and start of the spectral recording) and gate width g (time window for the spectral recording) were optimized for each sample and are specified in the captions of the respective spectra. The pulse frequency of the flashlamp was adjusted to match the full recording window (time between pulses $\geq d + g$).

The excitation and emission spectra were corrected using the standard corrections supplied by the manufacturer for the spectral power of the excitation source and the sensitivity of the detector. The phosphorescence lifetimes were measured using a μF2 pulsed 60 W Xenon microsecond flashlamp, with a repetition rate of 0.25 Hz and time-gated detection with a multichannel scaling (MCS) module. The emission was collected at right angle to the excitation

source. For experiments at 77 K, an Oxford Instruments OptistatDN, controlled via an Oxford Instruments MercuryiTC, was introduced into the sample chamber.

Quantum yields were determined on a Hamamatsu Quantaurus system (model type C11347-11), equipped with a 150 W Xenon arc lamp.

Solid and Liquid phase photoluminescence spectra. Fluorescence emission, excitation, and fluorescence quantum yields (QY) of all samples in solid-state and dispersed in solvents were also additionally measured using an FS-5 spectrofluorometer (Edinburgh Instruments), equipped with the SC-30 integrating sphere module for accurate absolute QY determination. To evaluate phosphorescence QY, the hybrid material (R = 38) was first photoactivated under 365 nm UV illumination for several minutes to populate the triplet states. Immediately following excitation, the samples were rapidly immersed in liquid nitrogen to suppress thermal quenching and stabilize the phosphorescent emission. Phosphorescence QY was then promptly recorded under cryogenic conditions to capture the persistent luminescence. All QY analyses were conducted using Fluoracle software to ensure reliable spectral integration and data processing.

Nuclear magnetic resonance (NMR) spectroscopy. ^{1}H NMR spectroscopy was performed on digested samples using a Bruker Avance III spectrometer. The digestion involved a solution of 400 μL methanol-d_4 and 100 μL DCl/D_2O (35 wt.%). Data were collected using a relaxation delay of 20 seconds, with 128 k points and a sweep width of 19.8 ppm, achieving a digital resolution of 0.18 Hz. Post-acquisition processing included a line broadening of 1 Hz and two rounds of zero-filling, conducted using Bruker Topspin software. MestReNova (v14.2.0) was used for additional data analysis, with chemical shifts referenced to the residual proton signal of DMSO and reported relative to tetramethylsilane (TMS).

Nanoindentation test. Nanoindentation tests were conducted to determine the mechanical properties, including Young's modulus and hardness, of PVDF, PS, and PMMA films. These measurements were performed using an iMicro nanoindenter (KLA-Tencor) equipped with a Berkovich diamond indenter tip.

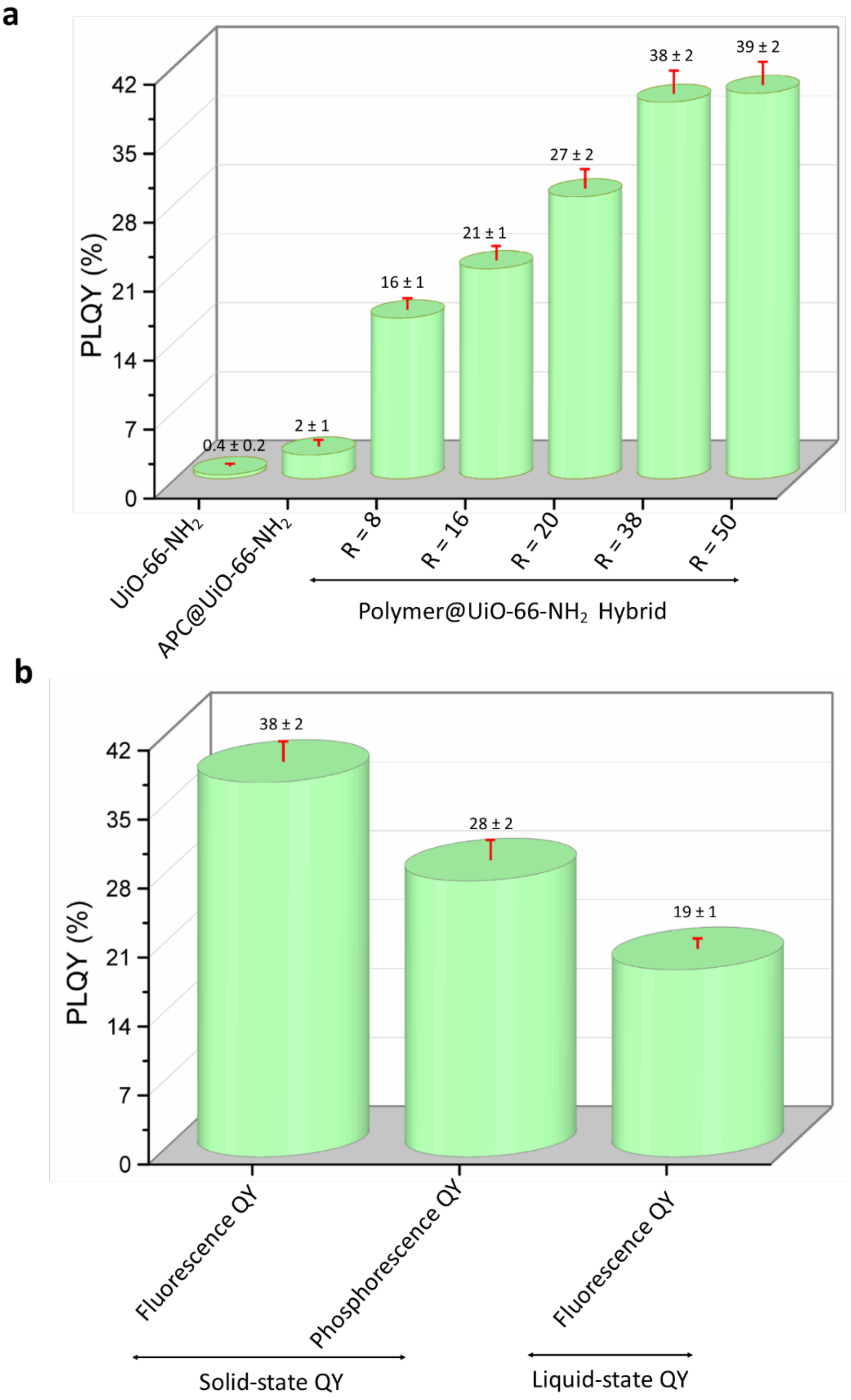

Fig. S1. (a) Fluorescence quantum yield (QY) of different materials, including various hybrids with varying R (ratio of HMDA: APC@UiO-66-NH_2) in solid-state. The PLQY reaches almost a constant value upon reaching R ≥ 38. (b) QY comparison of the hybrid material with a polymerization ratio of R = 38 in various states: fluorescence QY in solid powder form, phosphorescence QY in both the solid powder and the hybrid embedded within a PMMA

matrix, and QY in the aqueous (liquid) phase. This study utilized hybrid samples with R = 38 for all characterization and application studies.

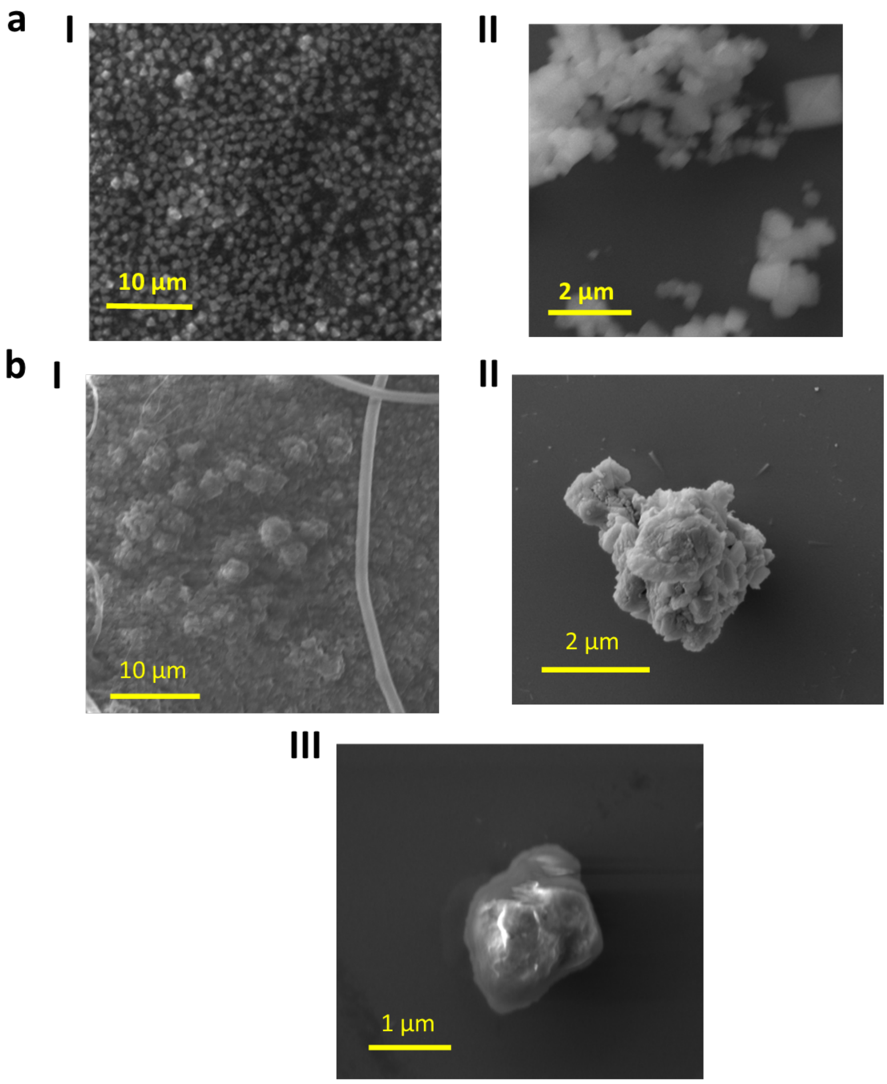

Fig. S2. SEM micrographs of pristine UiO-66-NH_2 MOF and hybrid (R = 38). (a) (I) Large area and (II) zoomed-in view of the SEM image of UiO-66-NH_2. The SEM image of UiO-66-NH_2 is diamond-shaped. (b) (I) Large area, (II) and (III) zoomed-in views of the SEM image of the hybrid composite. It is observed that the UIO-66-NH_2 morphology changes upon polymerization around it.

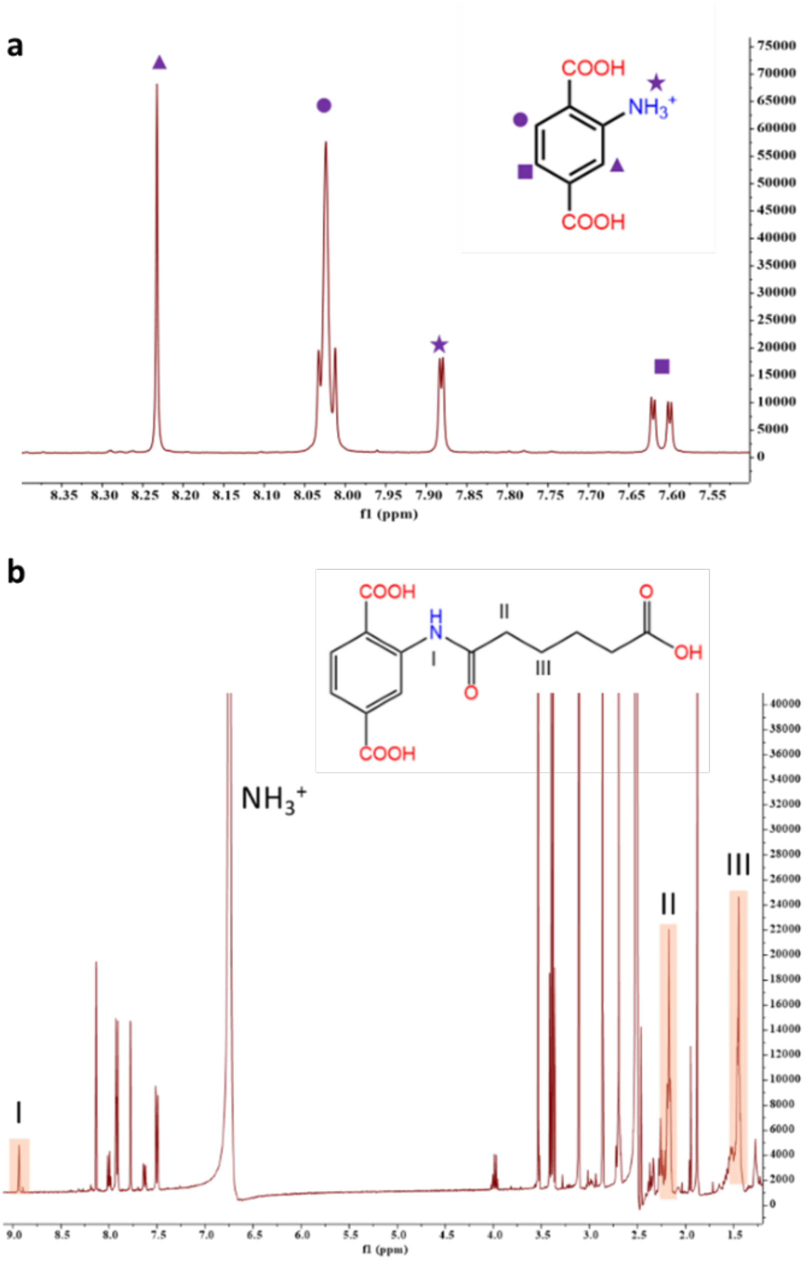

Fig. S3. ^{1}H NMR spectra of digested samples. (a) Pristine UiO-66-NH_2 MOF, (b) APC@UiO-66-NH_2. Highlighted regions correspond to specific proton environments in the chemical structures.

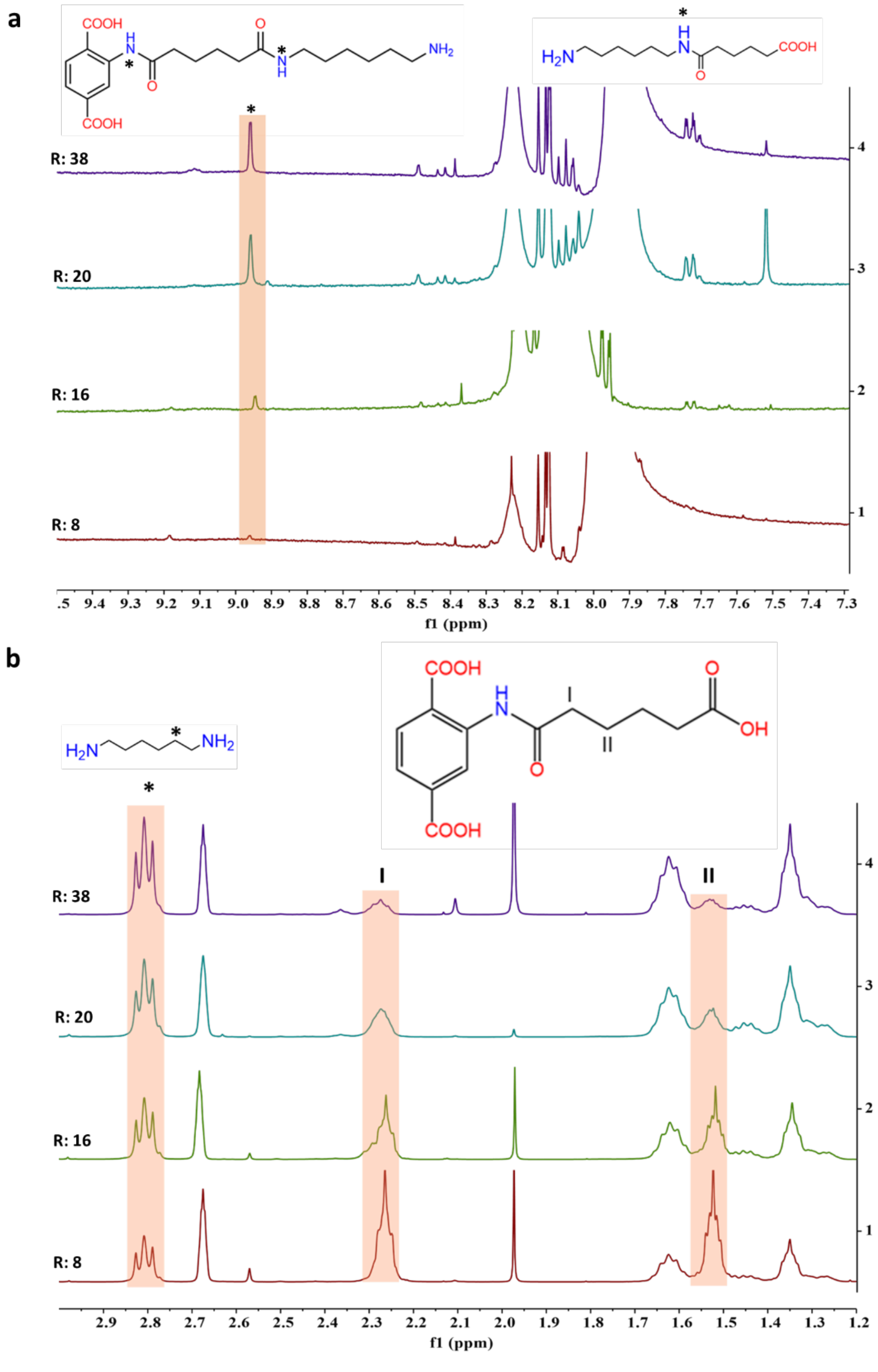

Fig. S4. ¹H NMR Analysis of Polymer/UiO-66-NH_2 Hybrids at Varying R Ratios. (a) ¹H NMR spectra highlighting peaks corresponding to amide bonds formed via the condensation of amines and carbonyl or acid groups from APC linkers. The intensity of amide-related peaks increases progressively as R changes from 8 to 20, reaching saturation at R = 20, where all APC carbonyl groups have reacted with HMDA. (b) ¹H NMR spectra emphasizing peaks associated with -CH_2 groups from HMDA and APC@UiO-66-NH_2. The intensity of peaks at 2.3 and 1.5 ppm decreases due to the reduced spin activity of APC CH_2 groups, which are

increasingly shielded by excess HMDA. Conversely, the peak intensity at 2.8 ppm rises with increasing HMDA concentration, reflecting its predominance in the hybrid system.

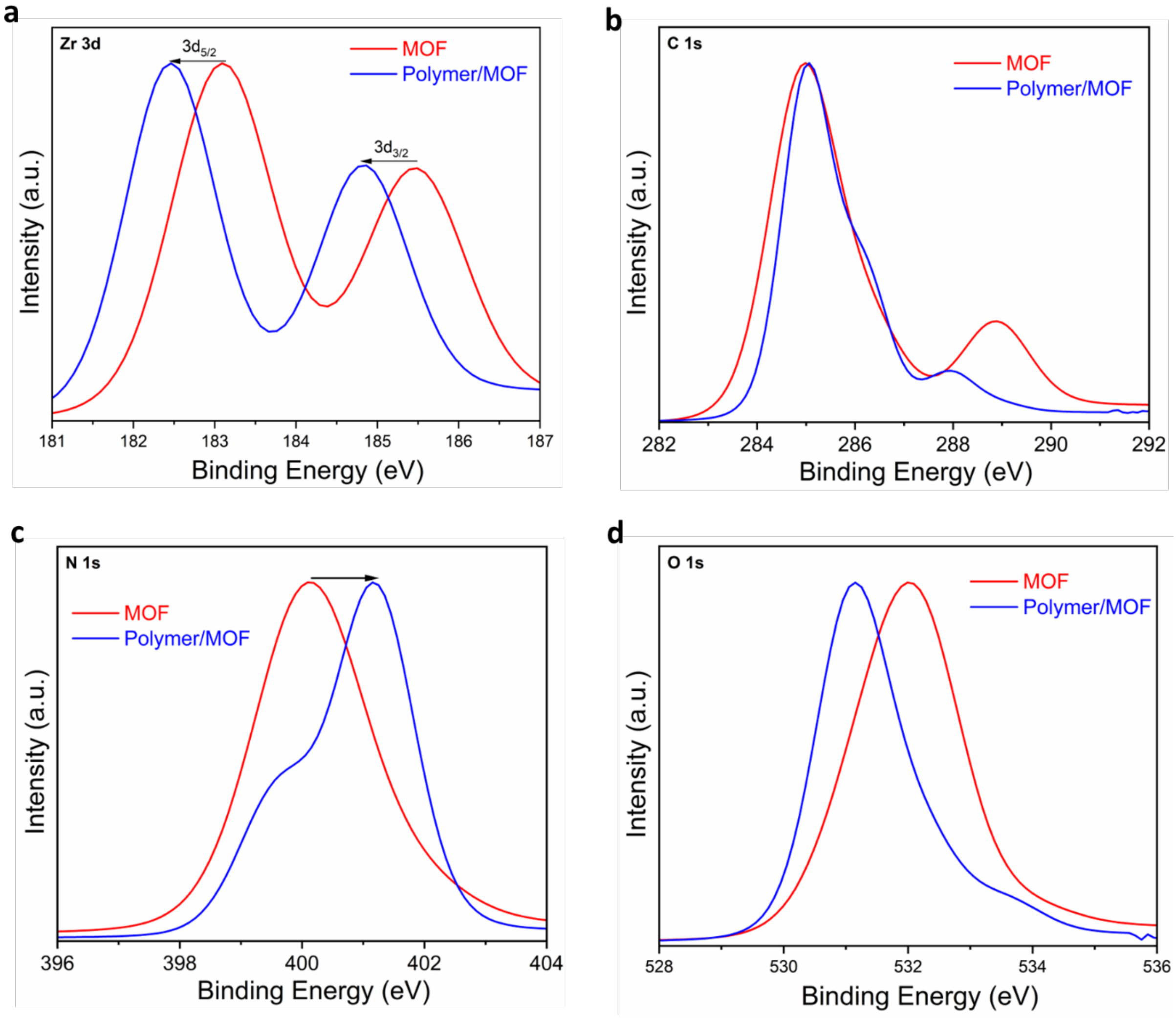

Fig. S5. High-resolution XPS spectra of MOF and hybrid (R = 38). XPS spectra for (a) Zr 3d, (b) C 1s, (c) N 1s, and (d) O 1s. The formation of the hybrid is evidenced by peak shifts and the emergence of new peaks, indicating covalent bonding between MOF amines and APC carbonyl groups.

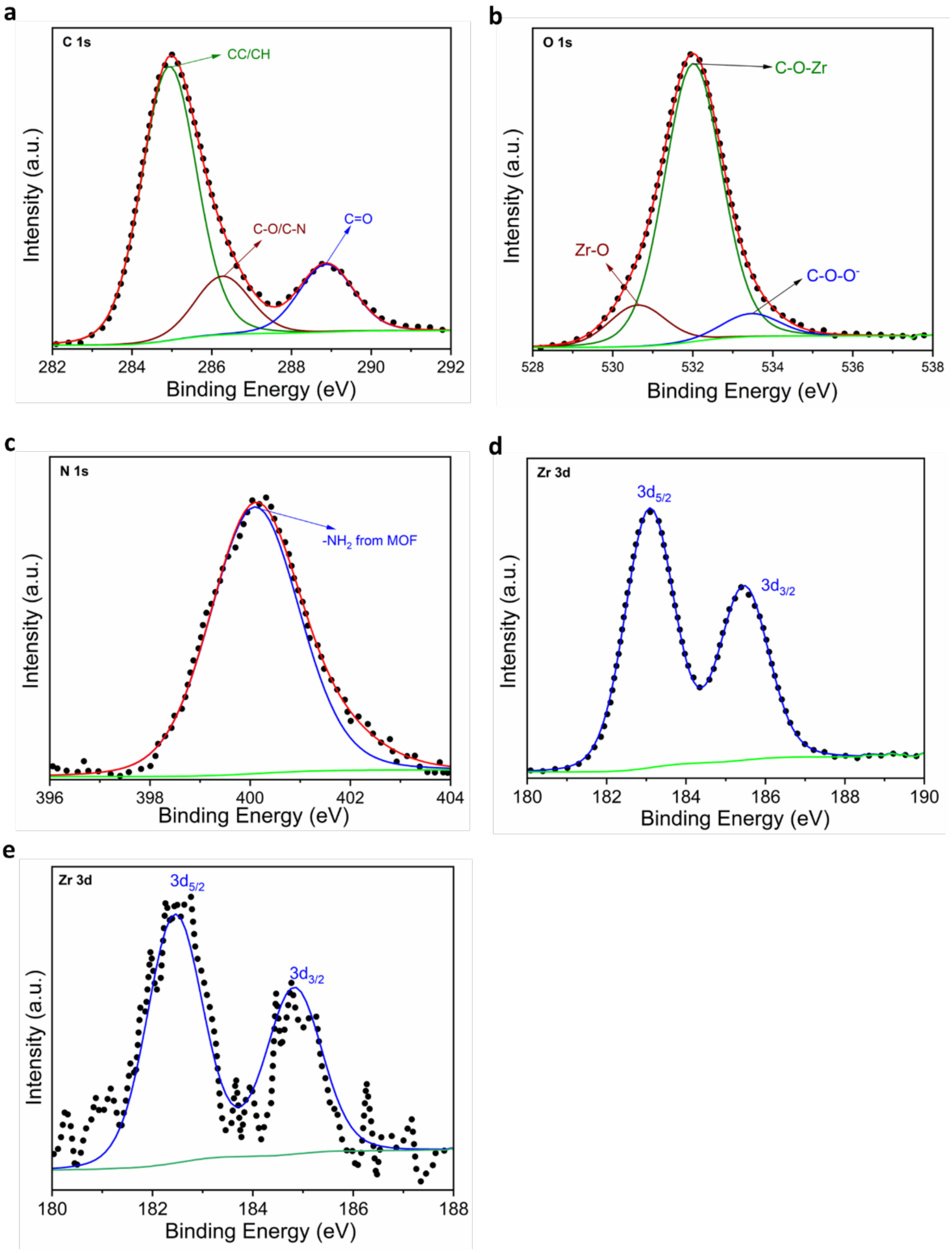

Fig. S6. High-resolution XPS of UiO-66-NH_2 MOF and hybrid (R=38). (a) C 1s, (b) O 1s, (c) N 1s, (d) Zr 3d, (e) Zr 3d of hybrid (R= 38).

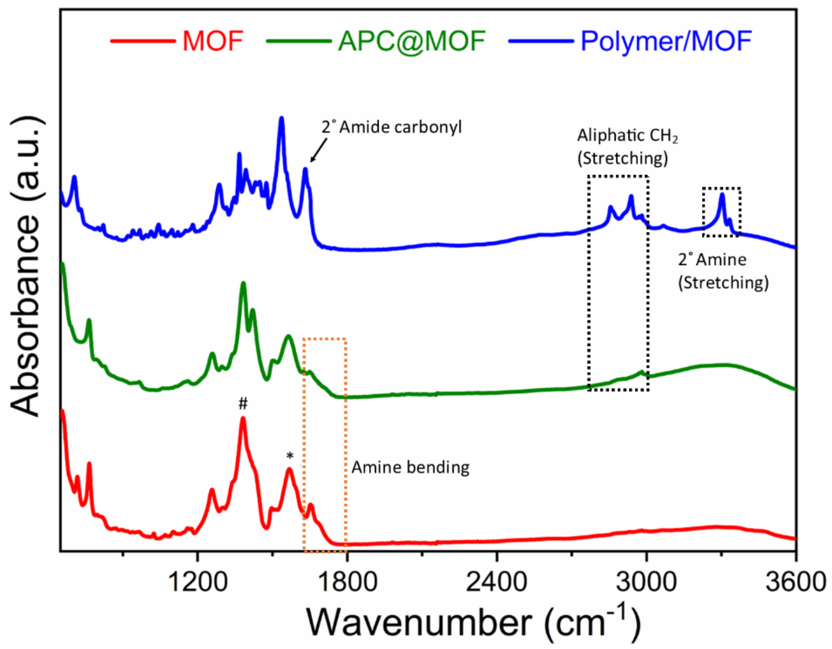

Fig. S7. FTIR spectra of pristine UiO-66-NH_2 MOF, APC@UiO-66-NH_2 (APC@MOF) and polymer/UiO-66-NH_2 (Polymer/MOF) with R =38. Characteristic peaks for the MOF (#) and hybrid materials are highlighted, with the dark yellow region indicating MOF aromatic amine bending and black regions showing polymer-specific peaks. The secondary amide carbonyl peak confirms condensation between amines and APC linkers.

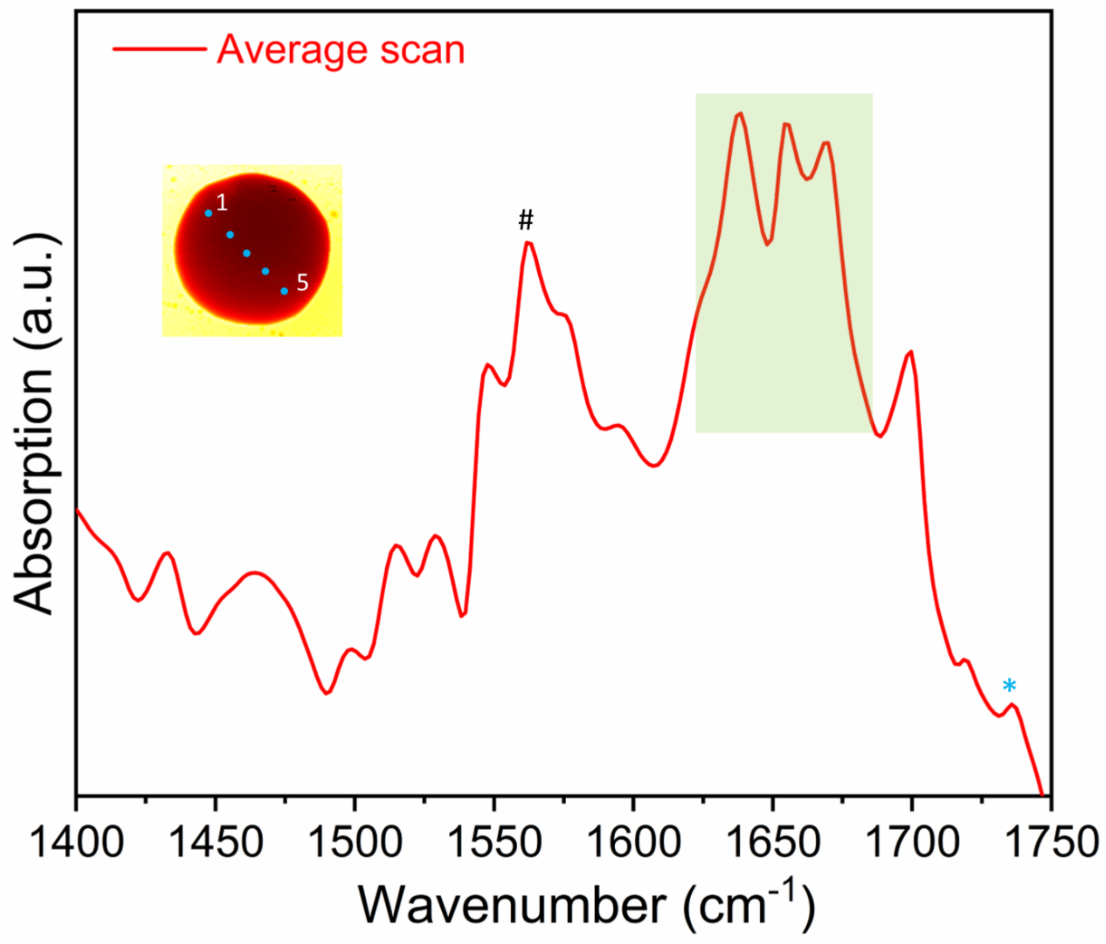

Fig. S8. Average nanoFTIR spectrum of the polymer/UiO-66-NH_2 hybrid, derived from five scans across the highlighted region in the inset's white light image.

Notable peaks include: the signature peak of UiO-66-NH_2 near 1560 cm^{-1} (denoted by #), secondary amide peaks appearing around 1625 and 1670 cm^{-1} (pale green region), and free carboxylic acid appearing around 1735 cm^{-1} (denoted by *).

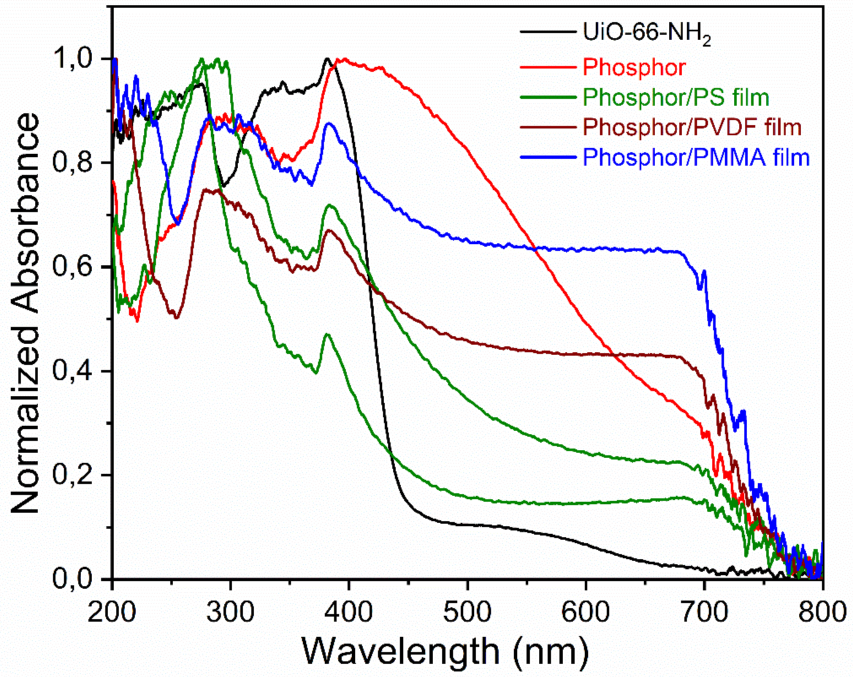

Fig. S9. UV-Vis absorption spectra of pristine UiO-66-NH_2 MOF, hybrid (phosphor), phosphor/PS film, phosphor/PVDF film, and phosphor/PMMA film obtained via diffuse reflectance spectroscopy (DRS).

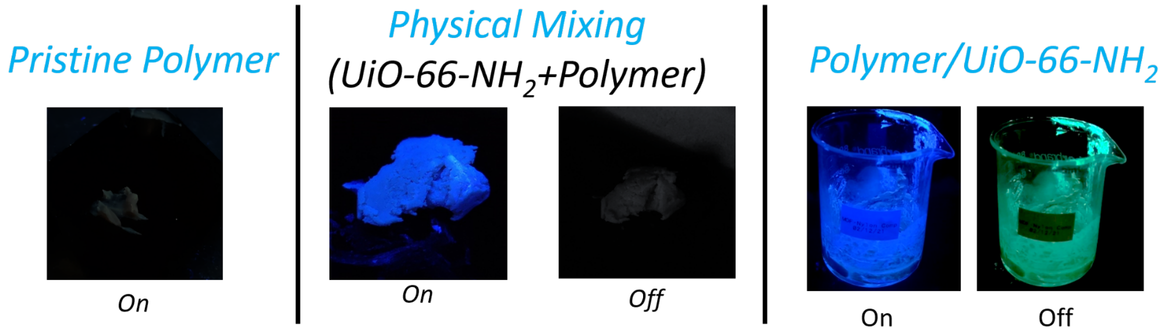

Fig. S10. Photographs under UV light (365 nm) showing pristine polymer and physical mixtures of polymer and UiO-66-NH_2 and polymer/MOF hybrid. The pristine polymer and physical mixture phase are non-phosphorescent in nature while the hybrid demonstrates phosphorescence.

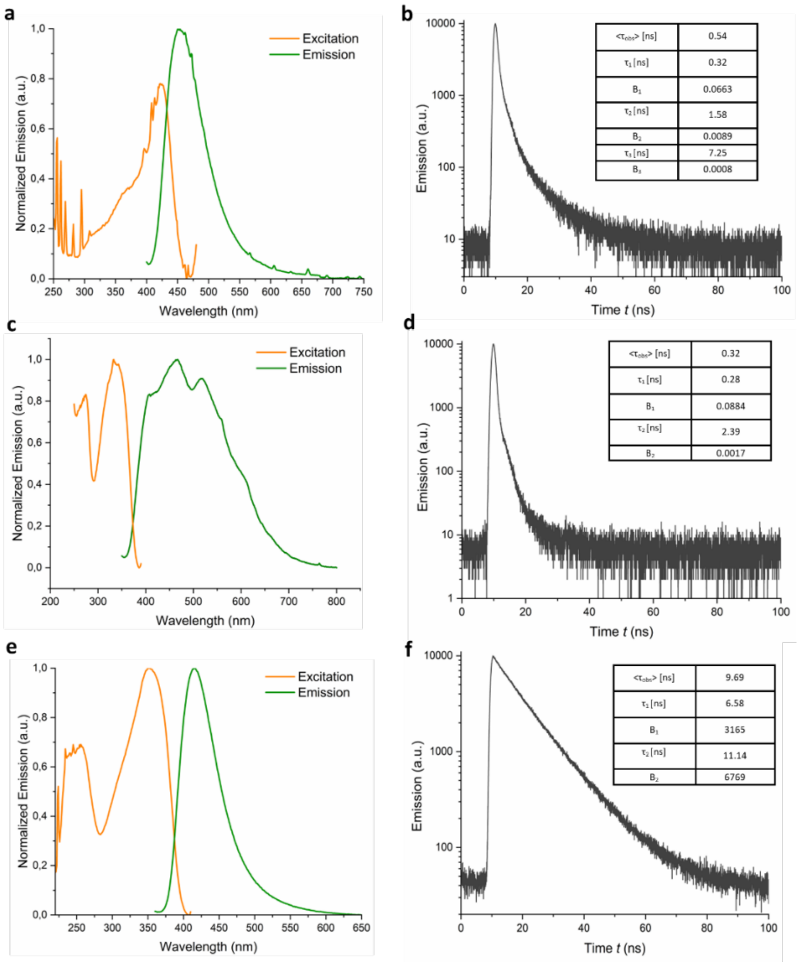

Fig. S11. Photoluminescence (PL) spectra of powder samples. PL spectra of pristine UiO-66-NH_2 (MOF), APC@MOF, and the hybrid (R = 38). Emission/excitation profiles and lifetime decay curves for (a, b) UiO-66-NH_2 (MOF), (c, d) APC@MOF, and (e, f) the hybrid (R = 38).

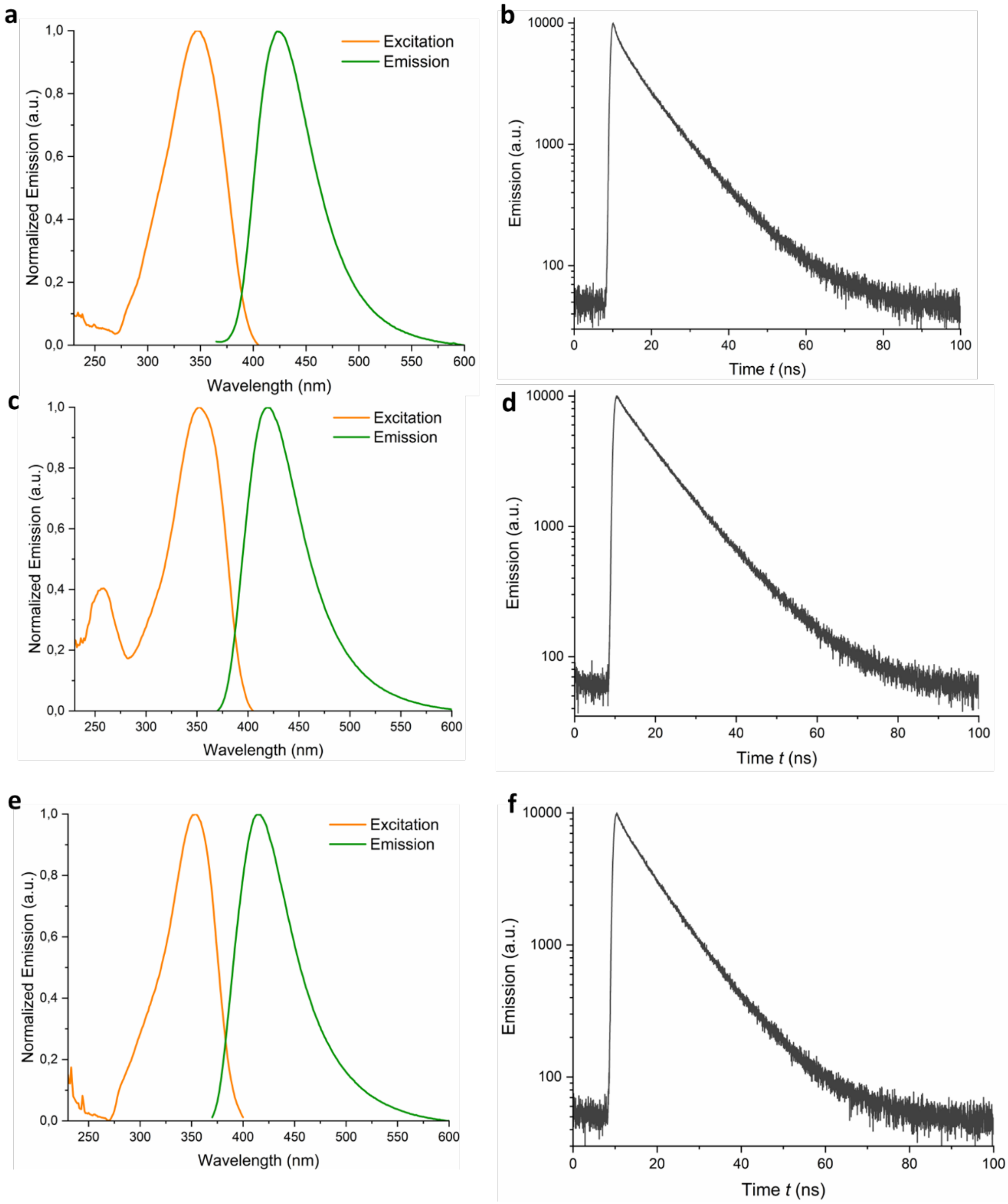

Fig. S12. PL properties of hybrid-embedded film samples. PL spectra and emission/excitation profiles of hybrid films embedded in PVDF, PS, and PMMA matrices. (a, b) Hybrid/PVDF, (c, d) Hybrid/PS, and (e, f) Hybrid/PMMA, including lifetime decay profiles.

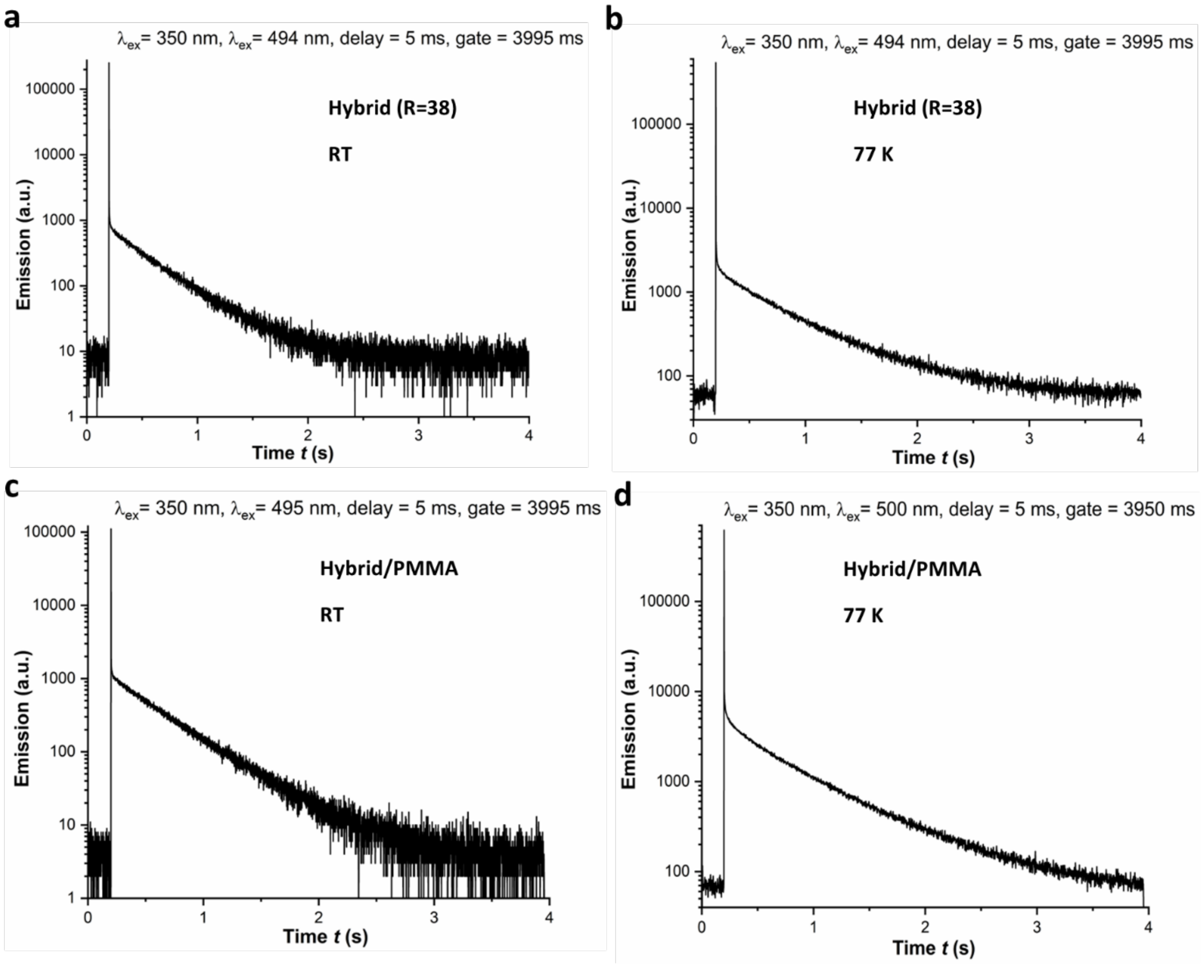

Fig. S13. Phosphorescence lifetime decay profile. Decay profile of Hybrid (R = 38) at (a) RT and (b) 77 K. Decay profile of Hybrid/PMMA film at (c) RT and (d) 77 K.

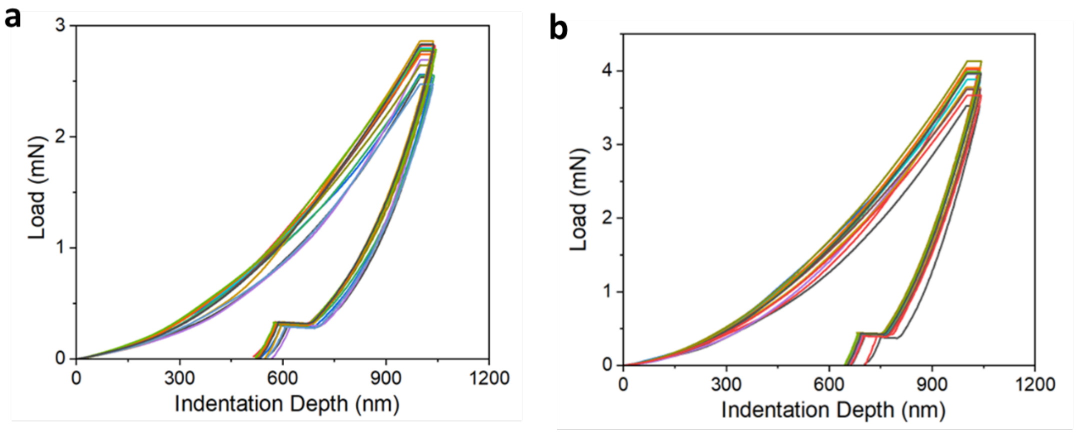

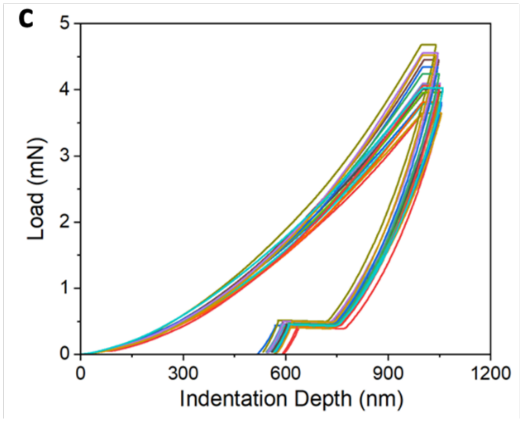

d

Polymer Matrices	Young Modulus (GPa)	Hardness (GPa)
PVDF	3.64	0.19
PS	4.41	0.23
PMMA	5.16	0.24

Fig. S14. Mechanical properties of pristine films. Load-depth curves from nanoindentation tests on pristine films of (a) PMMA, (b) PS, and (c) PVDF. (d) Table summarizing Young's modulus and hardness of the films, with all samples maintaining a consistent thickness of 380-400 μm.

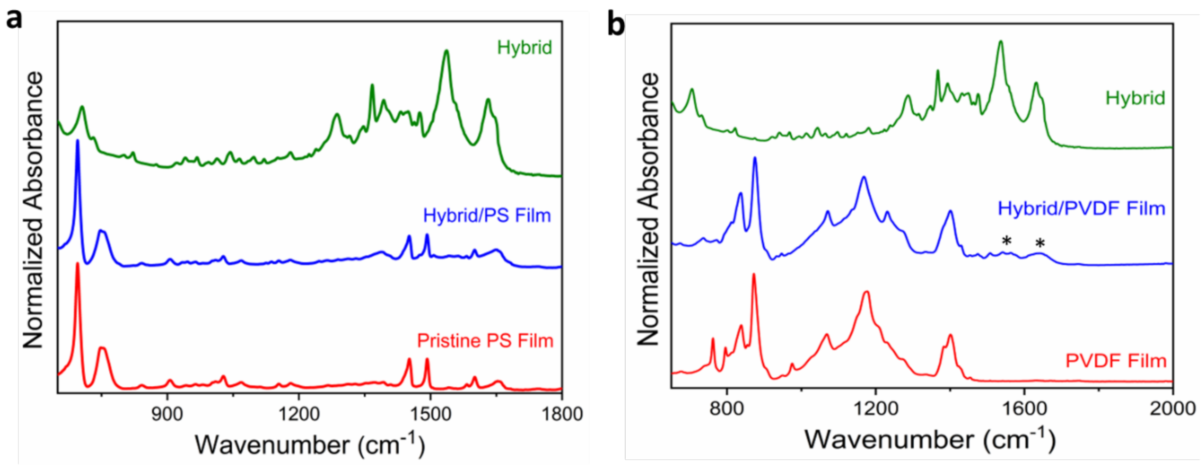

Fig. S15. FTIR of hybrid embedded films. (a) hybrid/PS and (b) hybrid/PVDF films. Highlighted peaks (*) represent the characteristic peaks for hybrid materials.

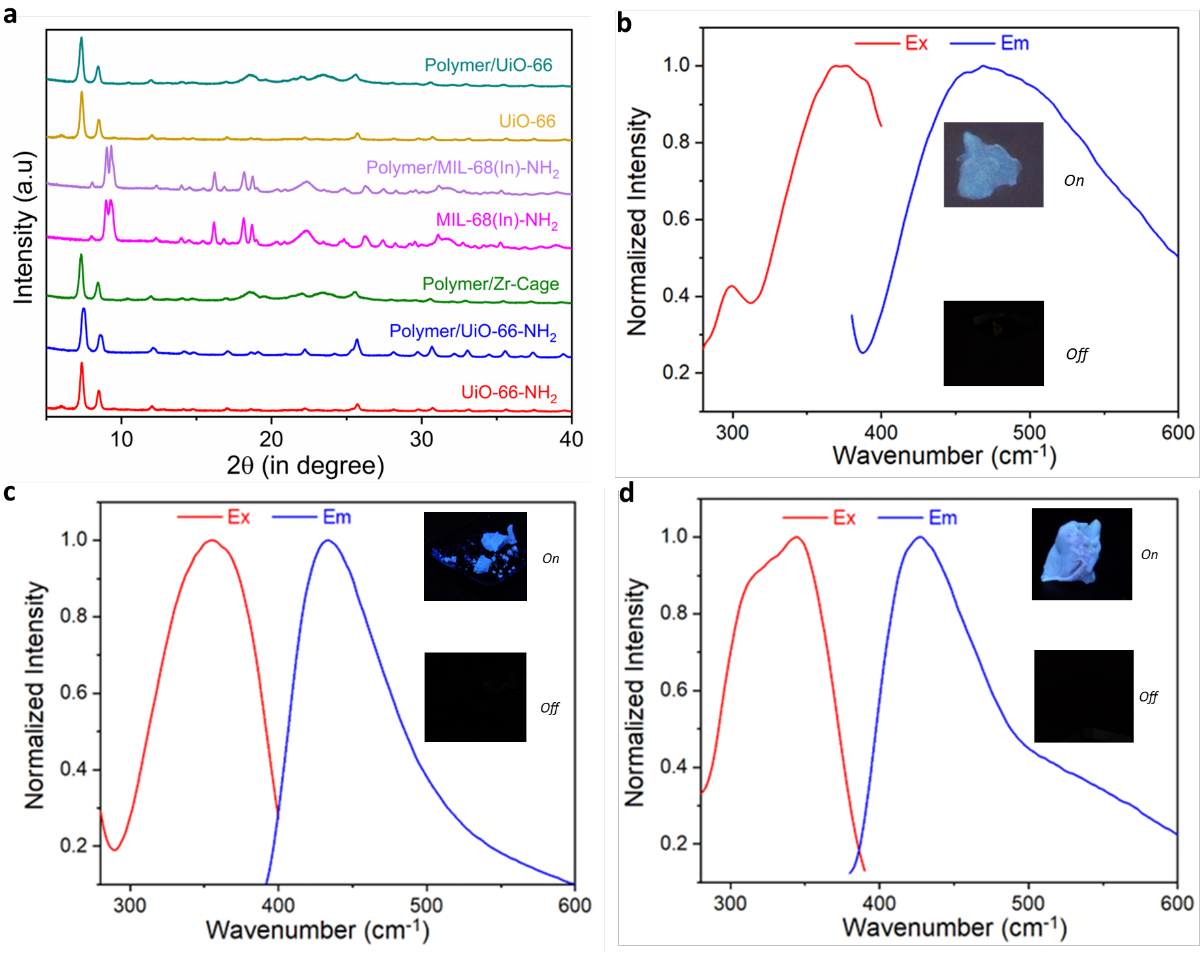

Fig. S16. (a) PXRD patterns of different hybrids (R= 38) constructed from various framework materials. PL spectra of (b) polymer/UiO-66 MOF, (c) polymer/Zr-Cage, and (d) polymer/MIL-68(In)-NH_2 MOF. Inset displaying photographs under 365 nm UV light (On) and without UV light (Off). The Ex and Em represent excitation and emission spectra, respectively.

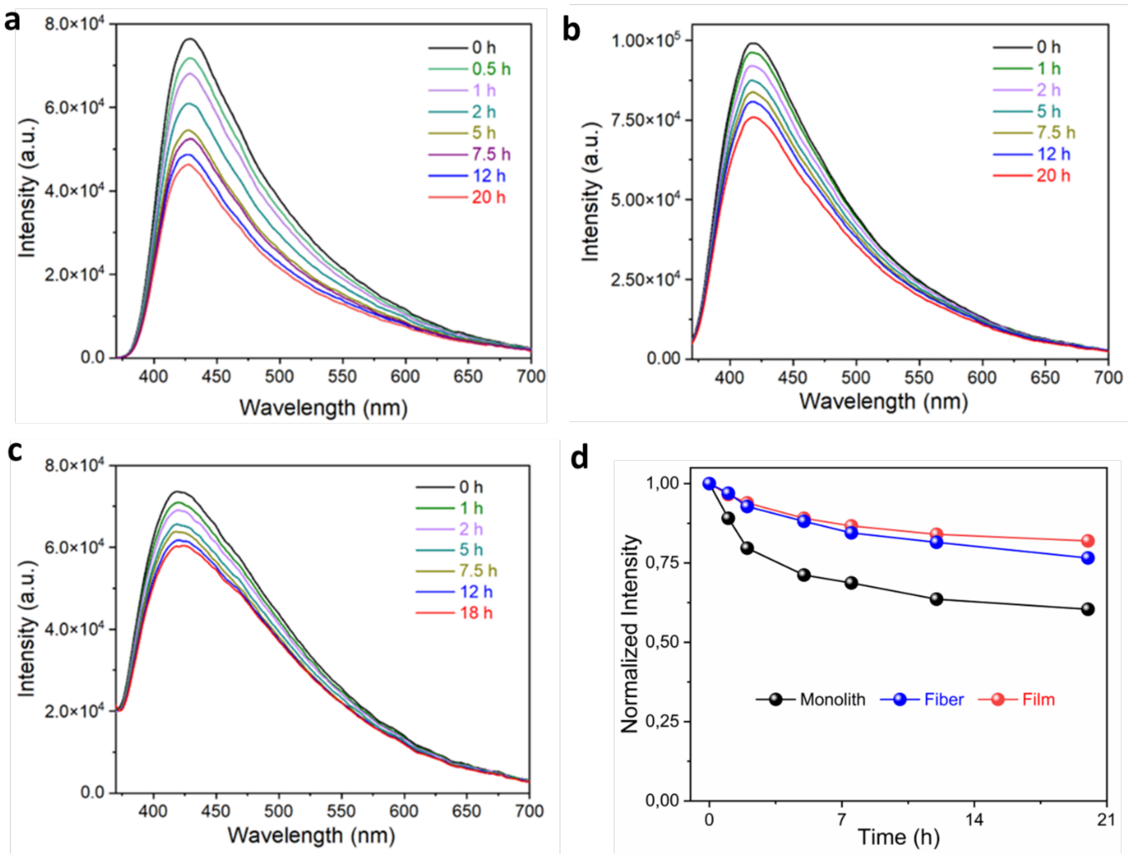

Fig. S17. Photostability tests for (a) hybrid monolith, (b) hybrid/PVDF fiber, (c) hybrid/PVDF film, and (d) comparison of photostability of monolith, hybrid/PVDF fiber and hybrid/film.

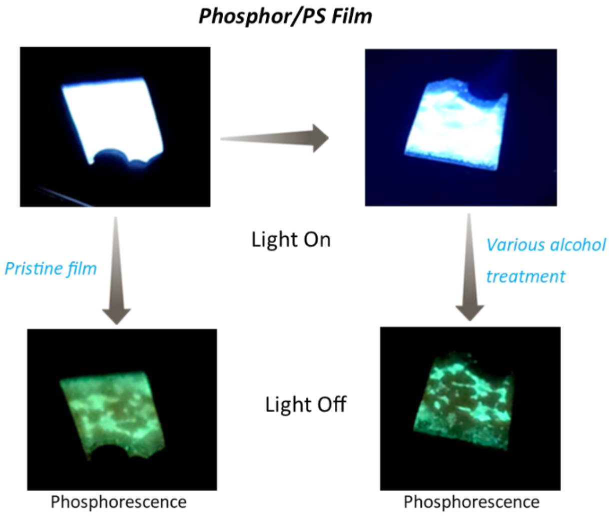

Fig. S18. Water-sensing using PS film embedded with polymer/UiO-66-NH_2 hybrid. Photographs of the PS film under UV light (365 nm), illustrating phosphorescence in its pristine state (absence of alcohol) and in the presence of various alcohols such as methanol, ethanol, isopropanol, and n butanol, indicating selective detection capabilities.

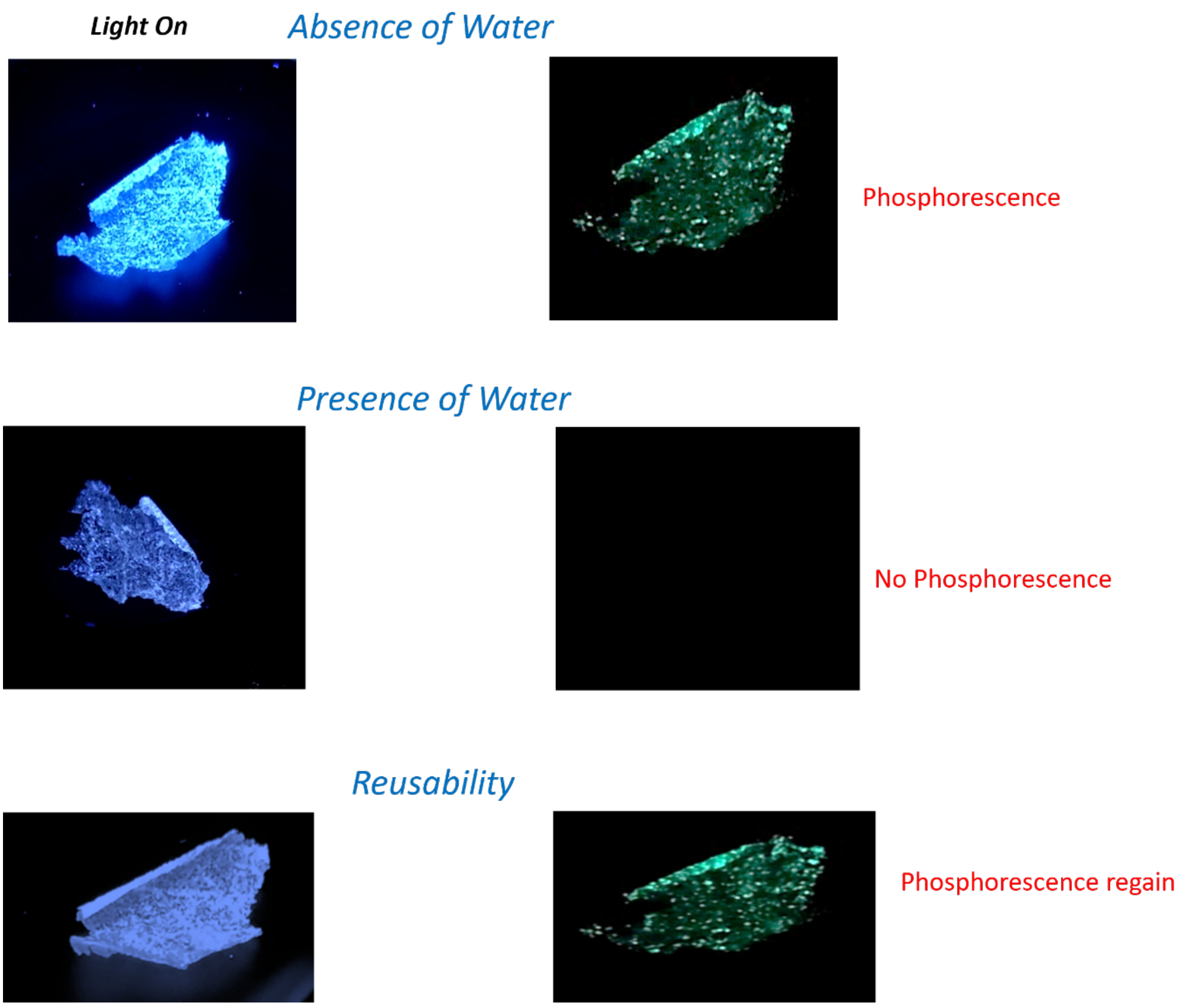

Fig. S19. Water-sensing using PVDF fiber embedded with polymer/UiO-66-NH_2 hybrid (R = 38). Photographs showing the phosphorescence behavior of the fiber sample under UV light (365 nm). The fiber exhibits phosphorescence in the absence of water, which disappears in the presence of water, demonstrating selective water-sensing capabilities. The fiber was also reused in the second cycle for water detection.

Movie Clips

Movie S1. *Phosphorescent emissions of hybrid-embedded PVDF under 365 nm UV light.* This clip reveals the phosphorescent behavior of a hybrid material embedded within PVDF film, excited by handheld 365 nm UV illumination.

Movie S2. *Phosphorescent emissions of hybrid-embedded PMMA under 365 nm UV light.* Video showing the striking phosphorescence behavior of hybrid embedded PMMA film, where the light was illuminated by handheld 365 nm UV light.

Movie S3. *Phosphorescent emissions of hybrid-embedded PVDF fiber under 365 nm UV light.* The video highlights the phosphorescent emission from hybrid-incorporated PVDF fibers when exposed to 365 nm UV light of a standard FS-5 spectrofluorometer.

Movie S4. *Phosphorescence in hybrid-embedded PS film (absence of methanol).* This clip showcases the phosphorescent response of a hybrid material in PS film in the presence of methanol solvent, illuminated by handheld 365 nm UV light.

Movie S5. *Phosphorescence in hybrid-embedded PS film (presence of methanol).* Video showing the phosphorescence behavior of hybrid embedded PS film presence of methanol solvent, where the light was illuminated by handheld 365 nm UV light.

Movie S6. *Phosphorescence in hybrid-embedded PS film (absence of water).* Experience the phosphorescent glow of a hybrid material embedded within PS film in the absence of water, activated by 365 nm UV light.

Movie S7. *Phosphorescence in hybrid-embedded PS film (presence of water).* This clip showcases the phosphorescent response of a hybrid material in PS film in the presence of water, illuminated by handheld 365 nm UV light.

Reference

1. S. Mollick, S. Saurabh, Y. D. More, S. Fajal, M. M. Shirolkar, W. Mandal and S. K. Ghosh, *Energy Environ. Sci.* , 2022, **15**, 3462-3469.
2. S. Mollick, S. Fajal, S. Saurabh, D. Mahato and S. K. Ghosh, *ACS Cent. Sci.* , 2020, **6**, 1534-1541.
3. G. L. Liu, Y. D. Yuan, J. Wang, Y. D. Cheng, S. B. Peh, Y. X. Wang, Y. H. Qian, J. Q. Dong, D. Q. Yuan and D. Zhao, *J. Am. Chem. Soc.*, 2018, **140**, 6231-6234.
4. S. Mollick, S. Rai, L. Frentzel-Beyme, V. Kachwal, L. Donà, D. Schürmann, B. Civalleri, S. Henke and J. C. Tan, *Adv. Sci.* , 2024, **11**, 2305070.
